# Acid- and Base-Mediated Hydrolysis of Dichloroacetamide Herbicide Safeners

**DOI:** 10.1021/acs.est.1c05958

**Published:** 2021-12-17

**Authors:** Monica E. McFadden, Eric V. Patterson, Keith P. Reber, Ian W. Gilbert, John D. Sivey, Gregory H. LeFevre, David M. Cwiertny

**Affiliations:** † Department of Civil and Environmental Engineering, University of Iowa, 4105 Seamans Center for the Engineering Arts and Sciences, Iowa City, Iowa 52242, United States; ‡ IIHR-Hydroscience and Engineering, University of Iowa, 100 C. Maxwell Stanley Hydraulics Laboratory, Iowa City, Iowa 52242, United States; § Department of Chemistry, Stony Brook University, 100 Nicolls Road, 104 Chemistry, Stony Brook, New York 11790, United States; ∥ Department of Chemistry, Towson University, Towson, Maryland 21252, United States; ⊥ Center for Health Effects of Environmental Contamination (CHEEC), University of Iowa, 251 North Capitol Street, Chemistry Building—Room W195, Iowa City, Iowa 52242, United States; # Department of Chemistry, University of Iowa, E331 Chemistry Building, Iowa City, Iowa 52242, United States

**Keywords:** dichloroacetamide safeners, safeners, hydrolysis, agrochemicals, pest control

## Abstract

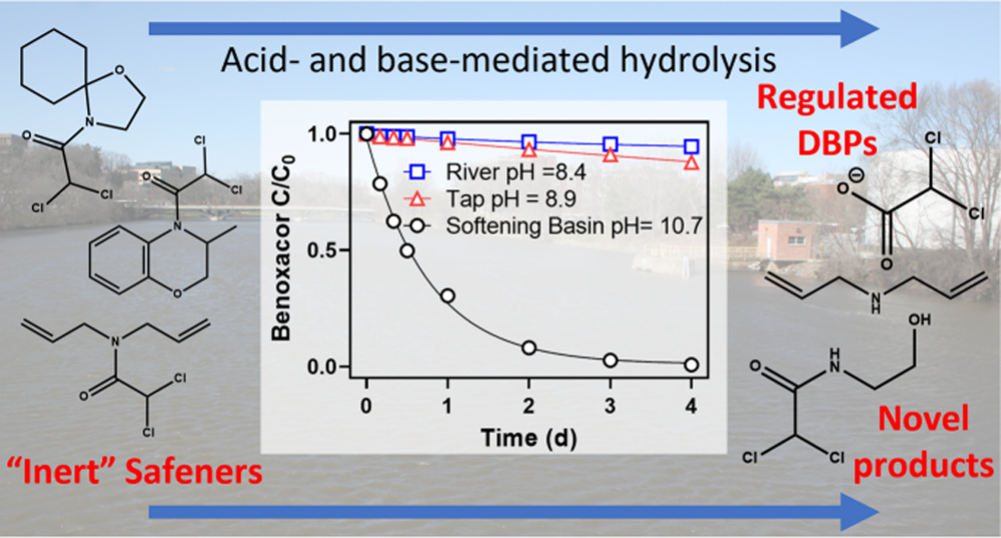

Safeners are used extensively in commercial herbicide formulations. Although safeners are regulated as inert ingredients, some of their transformation products have enhanced biological activity. Here, to fill gaps in our understanding of safener environmental fate, we determined rate constants and transformation products associated with the acid- and base-mediated hydrolysis of dichloroacetamide safeners AD-67, benoxacor, dichlormid, and furilazole. Second-order rate constants for acid- (HCl) and base-mediated (NaOH) dichloroacetamide hydrolysis (2.8 × 10^–3^ to 0.46 and 0.3–500 M^–1^ h^–1^, respectively) were, in many cases (5 of 8), greater than those reported for their chloroacetamide herbicide co-formulants. In particular, the rate constant for base-mediated hydrolysis of benoxacor was 2 orders of magnitude greater than that of its active ingredient co-formulant, *S*-metolachlor. At circumneutral pH, only benoxacor underwent appreciable hydrolysis (5.3 × 10^–4^ h^–1^), and under high-pH conditions representative of lime-soda softening, benoxacor’s half-life was 13 h—a timescale consistent with partial transformation during water treatment. Based on Orbitrap LC–MS/MS analysis of dichloroacetamide hydrolysis product mixtures, we propose structures for major products and three distinct mechanistic pathways that depend on the system pH and compound structure. These include base-mediated amide cleavage, acid-mediated amide cleavage, and acid-mediated oxazolidine ring opening. Collectively, this work will help to identify systems in which hydrolysis contributes to the transformation of dichloroacetamides, while also highlighting important differences in the reactivity of dichloroacetamides and their active chloroacetamide co-formulants.

## Abstract 2

Dichloroacetamide safeners are widely present in surface waters. We demonstrate their hydrolysis in natural and engineered systems and identify products of potential risk to the ecosystem and human health.

## 1 Introduction

**Figure 1:**
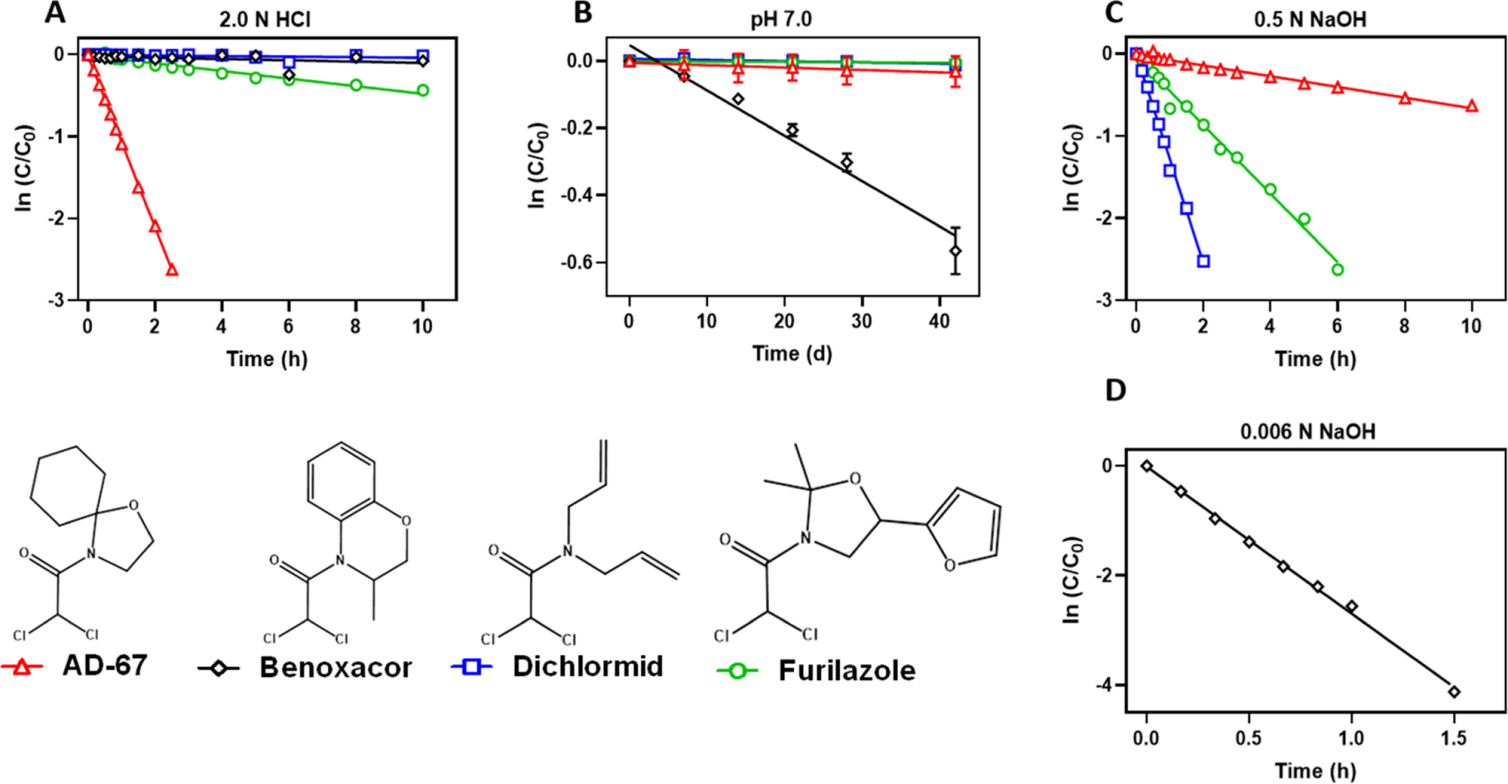
Natural logarithm of the normalized concentration (*C*/*C*_0_) of dichloroacetamide safeners in (A) 2.0 N HCl, (B) pH 7 phosphate buffer, (C) 0.5 N NaOH, or (D) 0.006 N NaOH as a function of time. Solid lines represent linear regression based on pseudo-first-order transformation kinetics. For (D), data are only shown for benoxacor at the relatively low NaOH concentration of 0.006 N to illustrate its relatively high reactivity in basic environments compared to the other dichloroacetamide safeners shown in (C) with 0.5 N NaOH. Rate constants for safener hydrolysis across a range of HCl (1–2.5 N) and NaOH (0.004–2 N) concentrations are provided in Table S5. Error bars indicating one standard deviation (*n* = 3) are present for the neutral conditions (B) but are smaller than some data points. Please note the differences in axis scales among the figure panels. Experiments were conducted at ambient temperature (22 ± 2 °C).

The dichloroacetamide safeners AD-67, benoxacor, dichlormid, and furilazole are commonly included in chloroacetamide herbicide formulations to selectively protect crops against herbicide toxicity.^1,2^ Dichloroacetamides (Figure 1) are modestly hydrophilic (log *K*_ow_ 1.84–3.19) and mobile in aqueous environments, which has led to their detection in Midwestern drinking water sources at concentrations of up to 190 ng/L.^2−6^ Given their widespread use (estimated >8 × 10^6^ kg/year globally) and occurrence in the environment, there is a growing body of research regarding the environmental fate and effects of dichloroacetamides.^2,4−10^ Recent studies have demonstrated moderate acute toxicity of dichloroacetamides toward model freshwater fish species (LC_50_ values of 1.4–4.6 mg/L),^11,12^ and toxicity screening data available from the U.S. Environmental Protection Agency (EPA) indicate the potential for benoxacor to interact with multiple human nuclear receptors.^13^ The EPA has also identified AD-67 and furilazole as “likely to be carcinogenic to humans”.^12,14^ Furthermore, some dichloroacetamide safeners, including dichlormid and benoxacor, can transform under environmentally relevant conditions to yield products with increased biological activity that may pose even greater risks to the environment and human health. For example, abiotic reductive dechlorination of dichlormid and benoxacor can, respectively, yield the regulated herbicide allidochlor (*N*,*N*-diallyl-2-chloroacetamide, commonly known as CDAA) and a known degradate, monochloro-benoxacor, which is toxic toward insect larvae.^7,8,15,16^

One important route by which dichloroacetamide safeners may transform in natural and engineered environmental systems is hydrolysis. Several studies have examined the acid- and base-mediated hydrolysis of chemical classes that share key structural features with dichloroacetamides, including the chloroacetamide herbicides that are commonly co-formulated with dichloroacetamide safeners and chemicals containing oxazolidine moieties.^17−23^ Hydrolysis rates for chloroacetamide herbicides vary significantly among different chemical species, with half-lives at circumneutral pH ranging from weeks for propisochlor and alachlor to years for more common species including acetochlor and metolachlor.^17,21,22,24−26^ Indeed, hydrolysis has been identified as a mechanism critical for governing the long-term fate of chloroacetamide herbicides in environments such as shallow aquifers where chloroacetamides are relatively persistent and other transformation processes are unlikely.^18,27−29^ Nevertheless, the corresponding investigations for dichloroacetamides are lacking, and thus, our understanding of timescales and environments where hydrolysis may influence dichloroacetamide fate remains incomplete. For example, we recently documented the transformation of thiamethoxam, a neonicotinoid insecticide, *via* hydrolysis in a full-scale lime-soda softening basin (pH ∼ 10.6), highlighting the importance of considering hydrolysis in such environmentally relevant, higher pH systems.^30^

We anticipate that dichloroacetamide safeners will undergo hydrolysis and yield transformation products in a manner analogous to chloroacetamide herbicides,^31−33^ with concomitant implications for human and ecosystem health. For most chloroacetamides, base-mediated hydrolysis proceeds primarily through a well-characterized bimolecular nucleophilic substitution (S_N_2) reaction, resulting in substitution of chloride with hydroxide (OH^–^), although some chloroacetamides undergo base-mediated amide cleavage.^17^ Acid-mediated chloroacetamide hydrolysis results in cleavage of both amide and ether groups.^17^ Slight differences in the chemical structure, particularly the type of (alkoxy)alkyl substituent, can dramatically influence the extent of chloroacetamide reactivity and the reaction mechanism due to changes in steric hindrance.^17^ For oxazolidine-based compounds, hydrolysis proceeds through a two-step reaction: rapid acid-mediated ring opening to yield a cationic Schiff base, followed by a slower hydrolysis involving addition of water.^34^ Although some of these reactions may indeed proceed *via* acid/base catalysis, we anticipate that some reactions may consume acid or base; therefore, we refer to all reactions as being mediated, rather than catalyzed, by acids and bases.

Several products of chloroacetamide hydrolysis reactions are reported to possess toxic attributes. For example, 2-chloro-2′,6′-diethylacetanilide, the *N*-dealkylation product of acid- and base-mediated hydrolysis of alachlor and butachlor, is mutagenic and may bind to DNA.^17,31,35−37^ Products of both acetochlor (2,6-diethylaniline) and 2-chloro-*N*-methylacetanilide (*N*-methylaniline) are teratogenic; indeed, 2,6-diethylaniline demonstrates increased teratogenicity toward frog embryos compared to its parent compound and is a promutagen.^32,33,38,39^ Considering the structural and behavioral similarity of dichloroacetamide safeners and their chloroacetamide herbicide co-formulants, it is possible that dichloroacetamides could yield analogous products with similar environmental and health effects.

To the best of our knowledge, no peer-reviewed studies have yet evaluated the hydrolysis of dichloroacetamide safeners, and data provided in technical reports are limited in terms of conditions assessed and the experimental timeframe.^40,41^ Determining the timescales, products, and pathways of dichloroacetamide hydrolysis is necessary for understanding dichloroacetamide persistence and fate and their associated risks to human and ecosystem health. Here, we systematically evaluated the hydrolysis rates, products, and mechanisms for the most common dichloroacetamide safeners, AD-67, benoxacor, dichlormid, and furilazole, in acidic, basic, and neutral pH aquatic systems. We also determined the degree of transformation in environmentally relevant systems where hydrolysis may control dichloroacetamide fate (*e.g.*, a chemical softening basin of a drinking water plant and alkaline surface waters). Hydrolysis product formation was initially monitored using high-performance liquid chromatography with diode array detection (HPLC-DAD) and subsequently characterized *via* Orbitrap high-resolution mass spectrometry (MS). Findings from this study will better aid environmental fate and risk assessment of this widely used yet overlooked chemical class in agroecosystems.

## 2 Materials and Methods

### 2.1 Reagents

Dichloroacetamide safeners included in this study are AD-67 (technical grade, Nanjing Essence Fine-Chemical Co., CAS 71526-07-3), benoxacor (99.4%, Sigma-Aldrich, CAS 98730-04-2), dichlormid (>97.0%, TCI America, CAS 37764-25-3), and furilazole (99.6%, Fluka, CAS 121776-33-8). For each safener, a 10 mM stock solution was prepared in HPLC-grade acetonitrile (Fisher Scientific). Whenever possible, potential hydrolysis products were either purchased (diallylamine, 99%, Sigma-Aldrich, CAS 124-02-7) or synthesized [3-methyl-3,4-dihydro-2*H*-1,4-benzoxazine and 2-amino-1-(2-furyl)ethanol] as described in the Supporting Information. A full list of reagents is available in the Supporting Information.

### 2.2 Hydrolysis Experiments

All experiments were conducted in acid-washed amber glass vials either at ambient temperature (22 ± 2 °C), in a heated 30 °C benchtop water bath or in a refrigerated 2 °C water bath. Dichloroacetamide safeners were introduced to 25 mL of phosphate buffer (5 mM, prepared in deionized water, purified to 18.2 MΩ·cm) by spiking 25 μL (corresponding to 0.1% of the resulting total volume) of a stock solution containing a safener in acetonitrile to achieve an initial concentration of 10 μM. For neutral pH systems, triplicate experiments were conducted in 5 mM potassium phosphate buffer adjusted to pH 7 and maintained without mixing at ambient temperature for up to 6 weeks. Temperature and pH were monitored, and 1 mL aliquots were taken at pre-determined time points for analysis. Base-mediated hydrolysis experiments were conducted for each safener using at least three NaOH concentrations, ranging from 0.004 to 2 N without replicates, and were sampled at least seven times over the course of 1–4 h. Based on the NaOH concentrations used and the short experimental timescales, we do not anticipate significant reaction between NaOH and the borosilicate glass vials.^42−44^ Sample aliquots (0.5 mL) were taken using volumetric pipettes (Eppendorf; Hamburg, Germany) and neutralized with 0.5 mL of equal-strength HCl to quench the reaction prior to analysis. Acid-mediated hydrolysis experiments were conducted over the course of 1–120 h in 1–2.5 N HCl with at least two HCl concentrations and at least seven sampling time points for each safener, without replicates. Equivalent volumes of equal-strength NaOH neutralized the reaction in samples collected for analysis. We note that for acid-mediated reactions with benoxacor, sample quenching with NaOH resulted in some incidental, near-instantaneous transformation of benoxacor, which was found to be highly sensitive to strong bases. In this case, acid-mediated benoxacor samples were neutralized using 1:5 dilution into 50 mM phosphate buffer adjusted to pH 13. Neutral pH experiments (pH 7) were run in parallel to all acid and base experiments to ensure that no other losses in the system (*e.g.*, sorptive losses) were observed over time.

### 2.3 Determination of Hydrolysis Rate Constants

Pseudo-first-order rate constants (*k*_obs_) were determined for all systems by regressing the natural logarithm of the normalized parent safener concentration (*C*/*C*_0_; where *C* is the safener concentration and *C*_0_ is the initial safener concentration) over time. All systems where the slope was significantly different from zero (*p* < 0.05) yielded good model fits (*R*^2^ > 0.96). In our strong acid and strong base systems, [H^+^] and [OH^–^] were assumed to be constant throughout our experiments because they were present in high excess (1 × 10^6^– 2.5 × 10^6^ -fold) relative to the initial benoxacor concentration (10 μM). Considering a constant concentration of [H^+^] and [OH^–^], second-order rate constants for acid-mediated and base-mediated hydrolysis (*k*_H_ and *k*_OH_, respectively) and first-order rate constants for hydrolysis by water at neutral pH (*k*_N_) were quantified in these constant-pH systems
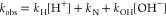
1where *k*_OH_[OH^–^] was assumed to be negligible in acidic (1–2.5 N HCl) and neutral (pH = 7) systems and *k*_H_[H^+^] was assumed to be negligible in basic (0.004–2 N NaOH) and neutral (pH = 7) systems.^45,46^ The second-order rate constants *k*_H_ and *k*_OH_ were determined by dividing experimental *k*_obs_ values by the assumed constant values of [H^+^] and [OH^–^], respectively, and then calculating the average and standard deviation of all experimental values (eqs S1 and S2). Uncertainties in hydrolysis rate constants are reported as standard deviations based on the average of acidic, basic, and neutral pH experiments.

### 2.4 Environmental Fate Studies

Benoxacor hydrolysis was also examined in environmental systems. Grab samples were collected from the University of Iowa Drinking Water Treatment Plant (UI DWTP) lime-soda softening basin. Iowa River water was collected from the UI DWTP post-screening. Tap water samples were collected from a laboratory tap at Seamans Center (University of Iowa, Iowa City, IA) after flushing the faucet for at least 2 min. All samples were sterile-filtered through 0.2 μm polystyrene bottle-top filters (Corning, Corning, NY) immediately following collection. Endogenous safener concentrations were assessed by analyzing unspiked samples *via* HPLC, but there were no detections. Samples were spiked with a stock solution containing a safener within 48 h after collection. Temperature and pH were monitored throughout experiments using a mercury thermometer and a pH meter (Fisher Scientific, Pittsburgh, PA). More information about UI DWTP processes and associated hydraulic residence times is provided in Table S1.

### 2.5 Analytical Methods

Safener concentrations and any detectable product formation were monitored *via* HPLC-DAD using our previously published methods.^7^ Orbitrap MS was used for accurate mass identification and MS/MS fragmentation to probe product structures. To aid in transformation product identification, samples were spiked with commercially available or synthesized authentic reference materials, when available. We used the Schymanski framework to communicate confidence in identifying products.^47^ Analytical methods are further described in the Supporting Information (Analytical Methods, Tables S2 and S3).

## 3 Results and Discussion

### 3.1 Dichloroacetamide Hydrolysis at pH 7

Under circumneutral conditions (pH 7.0 ± 0.1), only benoxacor hydrolyzed over a timescale [half-life of 55.0 (±3.7) days] that may be relevant to its environmental fate. Benoxacor hydrolysis at pH 7 followed first-order kinetics (Figure 1, Table 1). In contrast, AD-67, dichlormid, and furilazole did not transform by hydrolysis at pH 7.0 over the course of six weeks, indicating that these compounds would likely persist in near-neutral aqueous systems over long timescales without other transformation processes (*e.g.*, biotransformation, reductive dechlorination, and/or indirect photolysis; we have previously shown that these species are resistant to direct photolysis).^7^ See Table S4 for details on safener normalized concentrations, pH, and temperature throughout the experiment.

**Table 1:** Rate Constants for the Acid-Mediated, Base-Mediated, and Neutral Hydrolysis of Dichloroacetamide Safeners and Chloroacetamide Herbicides^a^

species	*k*_H_ (M^–1^ h^–1^)	*k*_N_ (h^–1^)	*k*_OH_ (M^–1^ h^–1^)	typical herbicide co-formulant
AD-67	0.46 ± 0.14	* (6 weeks)	0.30 ± 0.17	acetochlor
benoxacor	2.8 (±1.4) × 10^–3^	5.3 (±0.4) × 10^–4^	500 ± 200	metolachlor
dichlormid	* (5 days)	* (6 weeks)	2.9 ± 1.6	acetochlor
furilazole	3.1 (±0.7) × 10^–2^	* (6 weeks)	3.5 ± 1.8	acetochlor
metolachlor^b^	6 (±2) × 10^–4^	N/A	7.0 (±0.2) × 10^–3^	
acetochlor^b^	0.120 ± 0.008	N/A	1.35 ± 0.04	

a Rate constants were calculated in 5 mM phosphate buffer, either at pH 7 (for neutral conditions, *k*_N_), 1–2.5 N HCl (for acidic conditions, *k*_H_), or in 0.004–2 N NaOH (for basic conditions, *k*_OH_). Base-mediated hydrolysis experiments for benoxacor required dilute NaOH solutions. Experimental conditions are reported in Table S5. Asterisk (*) indicates a slope that was not statistically different from zero (value in parentheses is the duration over which samples were collected). N/A = data not reported.

b Herbicide data from Carlson *et al.*(17) Errors were determined as the standard deviation of calculated rate constants for triplicate samples (*k*_N_) or single samples across a range of [H^+^] or [OH^–^] values (*k*_H_ and *k*_OH_).

### 3.2 Dichloroacetamide Hydrolysis Kinetics in Acidic and Basic Model Systems

Dichloroacetamide safeners undergo acid-mediated and base-mediated hydrolysis in systems containing HCl (1–2.5 N) or NaOH (0.004–2 N) (Figure S1). In acidic systems, three of the safeners, AD-67, benoxacor, and furilazole, transformed at rates (*k*_H_ values from 2.8 × 10^–3^ to 0.46 M^–1^ h^–1^) consistent with their chloroacetamide counterparts (*k*_H_ values from 6 × 10^–4^ to 0.12 M^–1^ h^–1^),^17^ whereas dichlormid remained stable for over 5 days in 2 N HCl at 22 °C (Table 1). Under basic conditions, AD-67, benoxacor, dichlormid, and furilazole demonstrated similar or greater reactivity toward hydroxide (*k*_OH_ values range between 0.3 and 500 M^–1^ h^–1^) compared to their herbicide co-formulants, acetochlor and metolachlor (*k*_OH_ values 7.0 × 10^–3^ to 1.35 M^–1^ h^–1^),^17^ suggesting the potential for greater persistence of active herbicides compared to their safener co-formulants in alkaline environments (Table 1). Notably, the *k*_OH_ value for benoxacor is at least 2 orders of magnitude larger than that measured for the other safeners and reported for the chloroacetamide herbicides, illustrating that in basic environments, benoxacor is significantly more reactive than compounds with similar chemical structures.^17^ Half-lives for each safener are plotted according to pH in Figure S2 to provide an intuitive visual comparison of the reactivity across different dichloroacetamide species.

We attribute the greater reactivity of benoxacor toward hydroxide—even relative to other dichloroacetamide species—to the ability of the carbonyl carbon to lose electron density *via* both resonance and induction. Unlike the other dichloroacetamide safeners, the lone pair on the amide nitrogen of benoxacor is delocalized through the aromatic ring *via* resonance, significantly mitigating the resonance structure (*i.e.*, N=C–O^–^) that typically makes amides relatively unreactive toward nucleophilic attack.^48,49^ Moreover, relative to monochlorinated active ingredients such as metolachlor, benoxacor (like other dichloroacetamides) is anticipated to experience greater inductive withdrawal of electron density from its carbonyl carbon, further increasing the rate of nucleophilic attack at that location. Additional discussion of benoxacor’s reactivity can be found in the Supporting Information.

### 3.3 Benoxacor Hydrolysis in Environmentally Relevant Systems

**Figure 2:**
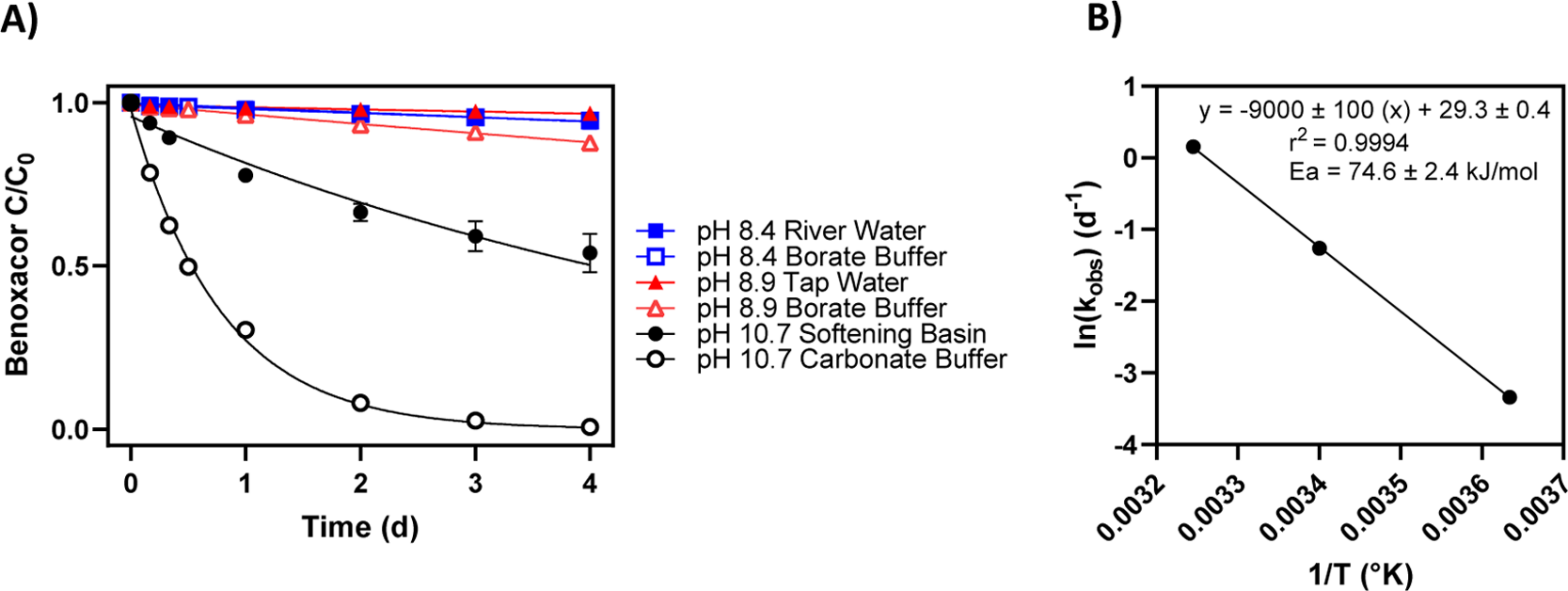
(A) Normalized concentration of benoxacor in tap water, Iowa River water, UI DWTP softening basin water, and sodium carbonate or sodium borate buffered systems (at indicated pH corresponding to environmental samples) as a function of time. Trendlines represent fitted exponential curves based on a pseudo-first-order model. Error bars indicate standard deviation among replicates (*n* = 3) but in some cases are obscured by the data symbol. (B) Arrhenius plot of benoxacor hydrolysis in pH 10.6, 5 mM phosphate buffer. The trendline represents the fitted linear regression; equation, coefficient of determination, and calculated activation energy (including its 95% confidence interval) are provided. In both figure panels, error bars indicate standard deviations (*n* = 2) and are smaller than the data point when not visible.

The UI DWTP operates its softening basin between pH 10.6 and 11.0; in our grab samples collected from the basin (initial pH of 10.71), we measured a half-life for benoxacor of 4.30 (±0.06) days, corresponding to an overall hydrolysis rate constant (*k*_obs_) of 6.7 (±1.6) × 10^–3^ h^–1^ (Figure 2). The pH of the spiked softening basin samples was closely monitored and decreased throughout the 4 day experiment (from 10.71 to 10.15), in turn decreasing the available hydroxide concentration and the concomitant reaction rate (Table S6). Because of this pH drift, we calculated hydrolysis rates and half-lives over shorter timeframes when the shift in pH was less pronounced. Over the first 8 h, the pH of the softening basin samples decreased from 10.71 (±0.03) to 10.57 (±0.02), and the *k*_obs_ for benoxacor was 1.42 (±0.02) × 10^–2^ h^–1^, corresponding to a half-life of 2.0 (±0.2) days. Over 24 h, the pH further decreased to 10.46 (±0.03), resulting in a *k*_obs_ of 1.0 (±0.7) × 10^–2^ h^–1^ and a half-life of 2.8 (±0.2) days (Table S7). These results demonstrate that the benoxacor hydrolysis rate is significantly influenced by the pH drift over time in our experiments (*p* = 0.002 for the hydrolysis rate calculated at 4 days and after 8 h and *p* = 0.009 for the hydrolysis rate calculated at 4 days and after 24 h). Incidentally, in softening basin samples that were not spiked with benoxacor, the pH only decreased to 10.64 (±0.01) over the entire 4 day experiment, suggesting that consumption of OH^–^ during the hydrolysis reaction likely drives the pH change (Table S8). Smaller decreases in pH could be attributed to other causes, for example, absorption of carbon dioxide from the atmosphere.

Previous studies measured safener concentrations in surface waters at levels that were substantially lower than our experimental systems. Although our average experimental starting concentration was 12.3 μM to facilitate quantification using HPLC-DAD, Woodward *et al.* detected maximum benoxacor concentrations in surface water at 190 ng/L (7.3 × 10^–4^ μM).^5^ Thus, it is unlikely that benoxacor concentrations in the environment would be high enough to drive significant pH change due to consumption of OH^–^ during benoxacor hydrolysis. As such, we would expect benoxacor half-lives more closely aligned with those calculated from *k*_OH_ values of buffered systems.

To examine benoxacor hydrolysis rates in constant-pH systems, we conducted the same experiment in a 5 mM sodium carbonate solution with sufficient buffering capacity to maintain a stable pH of 10.67 (Table S9). In these constant-pH systems, the calculated *k*_obs_ (0.050 ± 0.002 h^–1^) was significantly greater than in the softening basin systems (*p* = 1.5 × 10^–5^), with a significantly shorter half-life of only 0.57 (±0.03) days (*p* = 0.001). As a typical residence time in a softening basin is 1–3 h (Table S1), these results are consistent with partial (up to 20% of starting concentration) transformation of benoxacor in chemical softening basins, with a mixture of parent benoxacor and its base-mediated hydrolysis products being present in the process effluent. However, we note that the observed half-life in these constant-pH, carbonate buffered systems is still significantly longer (*p* = 0.002) than the expected half-life calculated using the *k*_OH_ value for strong base experiments in Table 1, from which the estimated half-life of benoxacor should be on the order of 2–4 h at pH 10.7 (eq S3).

We suspect that the rate of benoxacor hydrolysis may be affected by other system factors, such as ionic strength. Some have even suggested developing kinetic rate expressions based on activities, rather than concentrations, for reactions conducted in electrolyte solutions to account for ionic strength effects (see eq S4 and Table S10 for mean activity coefficients for HCl and NaOH).^50,51^ Accordingly, we have also provided hydrolysis rate constants calculated using H^+^ and OH^–^ activities in Table S5, although this approach did not result in better agreement with the rate constant observed in the carbonate buffered system. Others have also observed ionic strength effects during hydrolysis of structurally related compounds. For example, Carlson *et al.* reported that half-lives for chloroacetamide herbicides increased by 30% when the ionic strength of the buffered solution was doubled from 1 to 2 M.^17^ We observed a much stronger effect in our systems; when the ionic strength was increased from 6 mM in NaOH systems to 10.3 mM in the sodium carbonate buffer (only a 1.7-fold difference), the half-life for benoxacor in the carbonate system was 40–90 times longer than predicted from the *k*_OH_ value from NaOH systems (eq S5, Table S11). It is possible that dichloroacetamide safeners are more sensitive to changes in ionic strength compared to their chloroacetamide herbicide co-formulants, especially if intermediates formed during their base-mediated hydrolysis are anionic and stabilized by counterions in solution.

We also examined benoxacor hydrolysis in samples from the Iowa River and a laboratory tap (see Figure 2A). Like the softening basin samples, the pH of the spiked river and tap water decreased over time, thus slowing the rate of hydrolysis over the course of the experiment. Details and results from these experiments, including pH measurements and benoxacor concentrations over time, are provided in the Supporting Information (Table S6). To probe the behavior of benoxacor under these environmentally relevant pH conditions while keeping the pH constant, experiments were conducted in sodium borate buffered systems at the initial pH of the river and tap samples (8.4 and 8.9, respectively). Benoxacor half-lives were 52.9 (±4.5) and 22.5 (±1.9) days in pH 8.4 and pH 8.9 buffered systems, respectively, suggesting that in river and tap water systems where pH is stable, hydrolysis may play a role in the long-term fate of benoxacor (Tables S9 and S11).

Temperature-controlled studies demonstrated that the rate of benoxacor hydrolysis is strongly dependent on temperature within an environmentally relevant range, with significantly lower rates at 2 °C compared to 35 °C (*p* < 0.001). Hydrolysis kinetics for benoxacor were approximately fourfold faster at 35 °C compared to systems at 21 °C, which is consistent with prior studies on structurally related chloroacetamide hydrolysis (Figure S3, Table S12).^52,53^ Arrhenius relationships for base-mediated hydrolysis indicated a lower activation energy for benoxacor compared to chloroacetamides and other organohalide herbicides, consistent with the greater reactivity for benoxacor, specifically, and dichloroacetamide safeners, more generally, compared to their herbicide co-formulants (eq S6, Table S13).

### 3.4 Identification of Dichloroacetamide Hydrolysis Products

Base-mediated hydrolysis appears to occur by the same pathway for all four dichloroacetamide safeners (Schemes S1–S4, Figures S4–S9). For benoxacor, dichlormid, and furilazole, each safener yielded two major hydrolysis products, one of which was dichloroacetate, based on the accurate mass and chlorine isotope signature. The second products for benoxacor, dichlormid, and furilazole had accurate masses [M + H] of 150.0911, 98.0967, and 128.0707, respectively. Standards for these proposed hydrolysis products were either synthesized (for benoxacor and furilazole) or were commercially available (for dichlormid). In these cases, standard additions confirmed the product structures as Benox-149, Dich-97, and Furil-127 with level 1 confidence based on the Schymanski framework (Table 2).^47^ Dichloroacetate was also identified as a product of base-mediated AD-67 hydrolysis, although we were unable to detect any corresponding hydrolysis product using either ESI (+) or (−) modes. Nevertheless, the observation of dichloroacetate as a product of AD-67 base-mediated hydrolysis suggests that AD-67 transforms by the same pathway proposed above for the base-mediated hydrolysis of the benoxacor, dichlormid, and furilazole. We therefore propose AD-141 as an expected product of base-mediated hydrolysis (see Scheme S1 for more details).

**Table 2:**
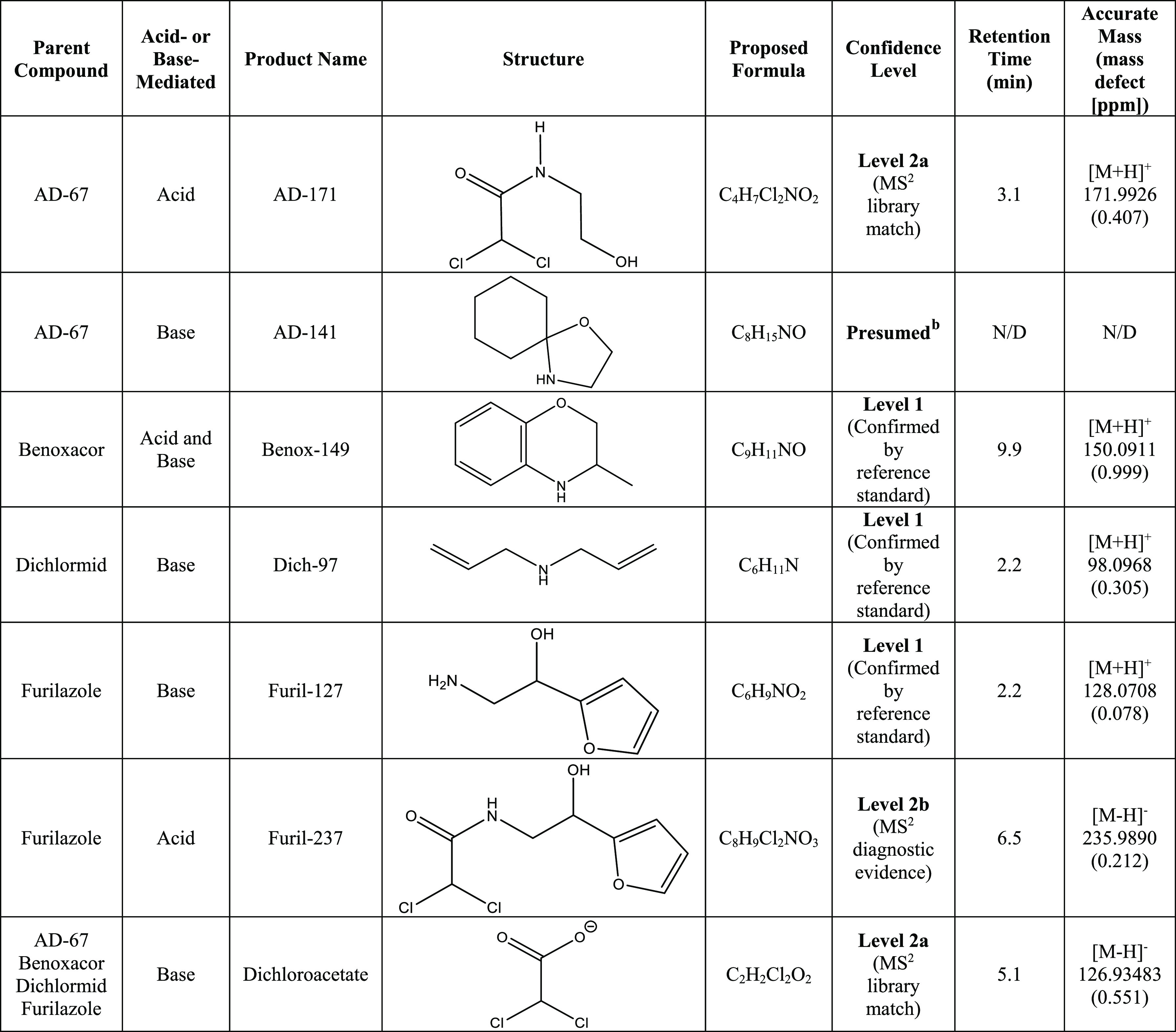
Hydrolysis Products of AD-67, Benoxacor, Dichlormid, and Furilazole^a^

a The confidence level of each product is described according to the framework outlined by Schymanski *et al.* for identifying small molecules *via* Orbitrap MS.^47^ Retention times correspond to reversed-phase LC–MS/MS experiments. MS data are presented in Figures S4–S11. N/D = not detected.

b Some structures were presumed to be present, despite no MS data indicating as such, due to the presence of dichloroacetate in the sample and established mechanisms for structurally similar compounds.

**Scheme 1:**
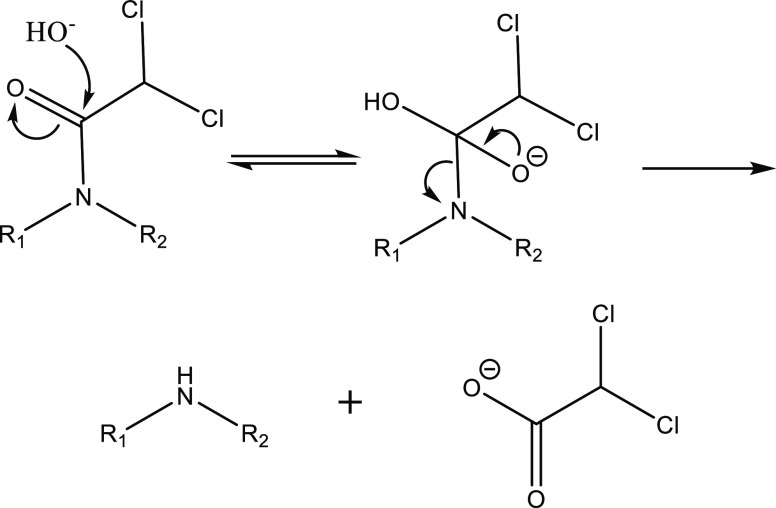
Base-Mediated Amide Cleavage Mechanism for AD-67, Benoxacor, Dichlormid, and Furilazole

Previous hydrolysis studies involving (mono)chloroacetamide herbicides yielded analogous products^17^*via* a base-mediated (B_AC2_) amide hydrolysis mechanism.^54^ This involves attack of the carbonyl carbon in the amide group by a hydroxide ion, resulting in the formation of an anionic tetrahedral intermediate that subsequently cleaves at the carbon–nitrogen bond (Scheme 1). We suspect that base-mediated dichloroacetamide hydrolysis proceeds *via* the same mechanism. Studies have also reported that the base-mediated hydrolysis of many chloroacetamide herbicides, particularly those with increased steric hindrance of the *N*-alkyl substituent that could slow the rate of OH^–^ attack at the amide carbon, proceeds through an intermolecular S_N_2 reaction at the chlorinated carbon center, resulting in the nucleophilic substitution of chloride by OH^–^.^17^ In our systems, there was no evidence in the MS data of hydroxy-substituted derivatives for dichloroacetamide safeners based on the anticipated exact masses for such products.

**Scheme 2:**
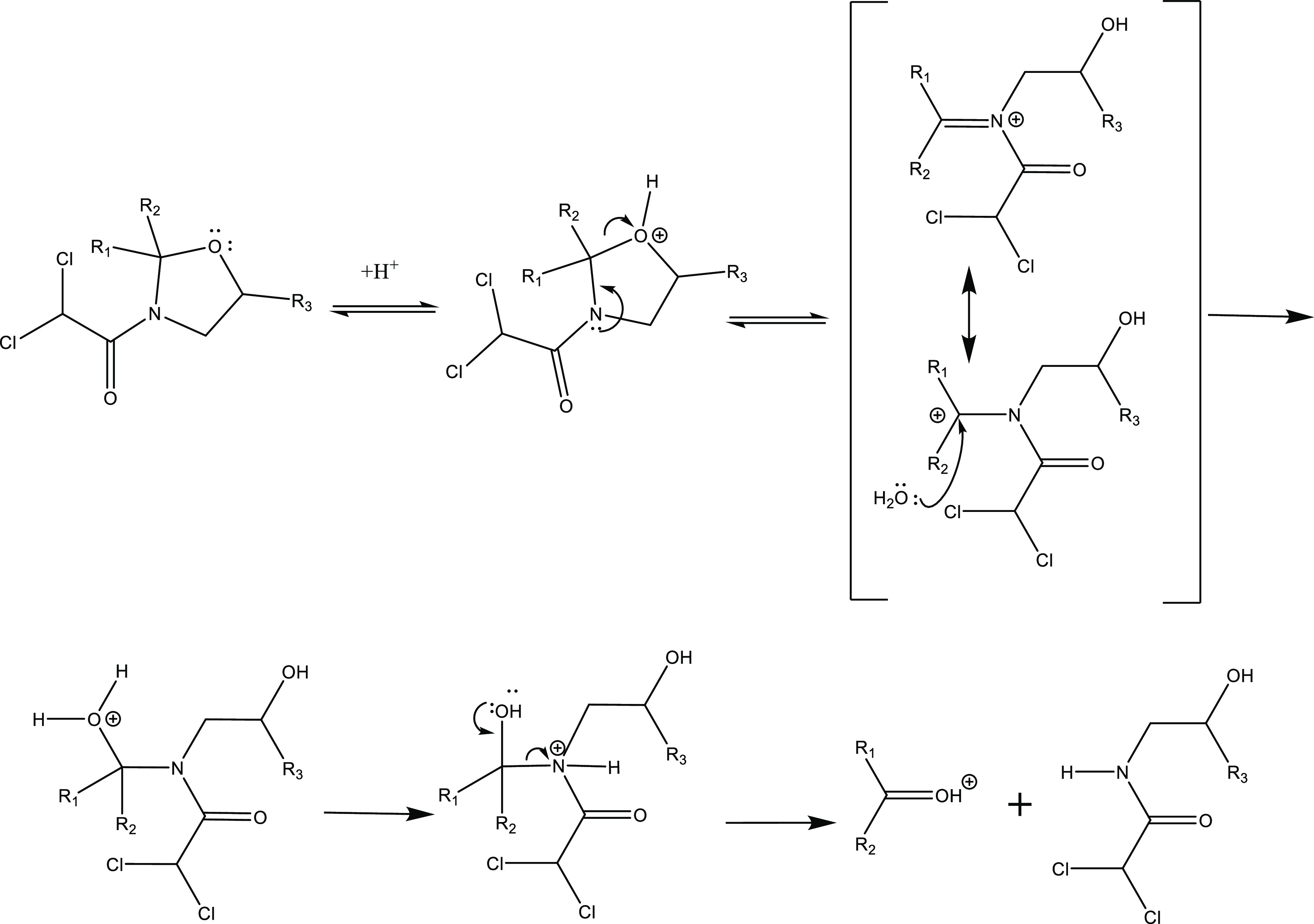
Acid-Mediated Hydrolysis Mechanism for Oxazolidine-Containing Molecules, AD-67 and Furilazole

**Scheme 3:**
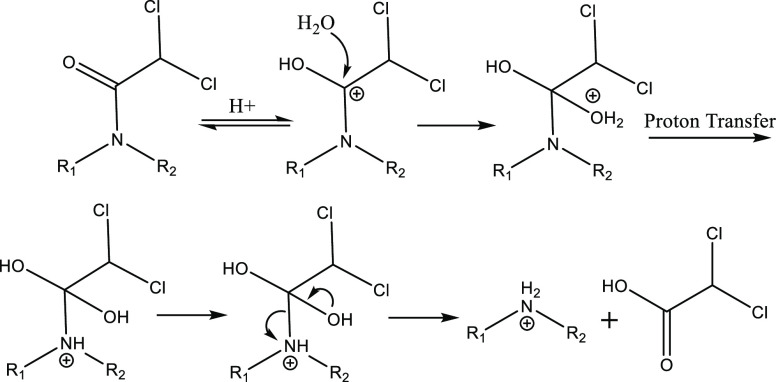
Acid-Mediated Amide Cleavage Mechanism for Benoxacor

For acid-mediated hydrolysis of AD-67 and furilazole, we detected products with [M + H] 171.9926 and 235.9890, respectively, which each possessed a dichloro isotope signature (Figures S10 and S11, Schemes S5–S7). Tentative product structures, AD-171 and Furil-237 (Table 2), suggest a well-known transformation mechanism involving the oxazolidine group that proceeds through an acid-mediated ring opening at the C–O bond to form a cationic Schiff base intermediate, followed by addition of water (Scheme 2).^55^ Previous studies have established that oxazolidines are highly sensitive to a wide range of pH values, and their reactivity depends greatly on the degree of electron delocalization by electron-withdrawing or electron-donating groups attached to the nitrogen of the five-membered ring.^55^ With benoxacor lacking an oxazolidine moiety, acid-mediated hydrolysis proceeded *via* a different mechanism. Benoxacor hydrolysis at low pH results in formation of the same benzoxazine derivative (Benox-149) and dichloroacetic acid that we observed as products of base-mediated hydrolysis. We propose that benoxacor proceeds through an acid-mediated amide cleavage mechanism similar to that which we proposed for base-mediated amide cleavage (Scheme 3). This mechanism has been previously reported for acid-mediated hydrolysis of chloroacetamide herbicides (Scheme S8).^17^ Dichlormid, in contrast to other dichloroacetamides, appears unreactive toward acid-mediated hydrolysis.

Hydrolysis products observed in this study may have important implications for water quality. For example, dichloroacetate is a regulated disinfection byproduct; the US EPA has established a maximum contaminant level for five haloacetic acids, including dichloroacetic acid, at 60 ppb. Derivatives of 1,4-benzoxazine are routinely used as bioactive scaffolds for the synthesis of anticancer, antibacterial, and antifungal pharmaceuticals.^56^ Such benzoxazine derivatives possess characteristics (*e.g.*, cell permeability, oral availability, *in vitro* stability, and straightforward synthesis) that suggest that they may act as prodrugs in mammals.^57−59^ Furthermore, all identified hydrolysis products elute from our reversed-phase column prior to their respective parent compounds, suggesting that the products are more polar and therefore likely more mobile than their parent in aqueous systems. Structural similarity to chloroacetamide derivatives may also suggest analogous toxicological effects to those determined for products of chloroacetamide hydrolysis.^17,31−33,35−38^ More work is needed to assess the fate and effects of dichloroacetamide hydrolysis products, especially those of benoxacor that are most likely to form in environmentally relevant systems.

### 3.5 Environmental Implications

This is the first study of which we are aware documenting dichloroacetamide safener hydrolysis in acidic, basic, and neutral environments. We determined that most dichloroacetamides transform by hydrolysis at rates that are similar to or greater than those of their respective chloroacetamide herbicide co-formulants. This is most notable for benoxacor, which we expect to undergo transformation by hydrolysis processes in some environmentally relevant settings (*e.g.*, water treatment) where its active ingredient, metolachlor, is persistent. The propensity for benoxacor to hydrolyze under alkaline conditions may be particularly relevant in parts of the Midwestern U.S., where benoxacor is extensively applied and the landscape is greatly affected by natural carbonate deposits, which can increase the pH and hardness (and thus require softening) of the surface and groundwater.

More generally, the rate constants determined in this study will be key for modeling the environmental fate of dichloroacetamide safeners that are widely used in agriculture and widespread in Midwestern surface waters.^5^ This new knowledge is important because hydrolysis may drive safener fate in some settings, given that as a class, dichloroacetamides are expected to undergo slow biological transformation.^2^ We have also developed generalizable insights into the acid- and base-mediated mechanisms of dichloroacetamide hydrolysis, which may prove useful both in assessing the fate and effects of their transformation products and predicting the transformation products of other structurally related agrochemicals.

There are also more practical implications for this work. For example, we demonstrate that hydrolysis can occur under conditions representative of those encountered during herbicide mixing and spraying, thus limiting the shelf life of dilute formulations containing safeners. Safener-containing formulations can be diluted with tap water or groundwater, which in the Midwest can have an elevated pH that promotes base-mediated hydrolysis. If it takes several hours in the spring for a farmer to spray a field, the high pH coupled with warm weather temperatures may diminish the efficacy of the safener. Furthermore, laboratory stock solutions and calibration standards containing benoxacor in an aqueous matrix may also be susceptible to transformation on timescales of a few weeks; thus, researchers should consider the implications of hydrolysis and recognize the value of preparing stocks in water-miscible organic solvents until the start of experiments.

Finally, we have provided another^30^ example in which the high pH encountered during chemical softening of drinking water can result in agrochemical transformation. Conditions used during chemical softening should be considered when assessing the potential for hydrolysis to influence the fate of emerging organic pollutant classes. We suspect that base-mediated hydrolysis in these systems may currently be overlooked as influencing pollutant fate during drinking water treatment. Moreover, base-mediated processes during chemical softening can also contribute to the formation of transformation products that may pose risks to public health if they persist through the distribution system.

## Supporting Information Available

Chemical synthesis data, analytical methods, rate calculations, activity calculations, ionic strength calculations, activation energy calculations, numerical results, high-resolution MS spectra, and proposed hydrolysis mechanisms (PDF)
