# Supplementary material for: Acid- and Base-Mediated Hydrolysis of Dichloroacetamide Herbicide Safeners
Source: Environ Sci Technol. 2021 Dec 17;56(1):325–34. doi: 10.1021/acs.est.1c05958 (PMC8733929; doi:10.1021/acs.est.1c05958)
Supplement: Supplementary file 1 — es1c05958_si_001.pdf [file es1c05958_si_001.pdf]

SUPPORTING INFORMATION

Acid- and Base-Mediated Hydrolysis of Dichloroacetamide Herbicide Safeners

Monica E. McFadden[†,‡], Eric V. Patterson[§], Keith P. Reber[‖], Ian W. Gilbert[‖], John D. Sivey[‖], Gregory H. LeFevre[†,‡], David M. Cwiertny*[†,‡,⊥,#]

[†]Department of Civil and Environmental Engineering, University of Iowa, 4105 Seamans Center for the Engineering Arts and Sciences, Iowa City, IA 52242, United States; [‡]IIHR-Hydroscience and Engineering, University of Iowa, 100 C. Maxwell Stanley Hydraulics Laboratory, Iowa City, Iowa, 52242, United States; [§] Department of Chemistry, Stony Brook University, 100 Nicolls Road, 104 Chemistry, Stony Brook, NY 11790, United States; [‖]Department of Chemistry, Towson University, Towson, MD 21252, United States; [⊥] Center for Health Effects of Environmental Contamination (CHEEC), University of Iowa, 251 North Capitol St., Chemistry Building – Room W195, Iowa City, IA 52242, United States; [#]Department of Chemistry, E331 Chemistry Building, University of Iowa, Iowa City, IA 52242

Prepared for *Environmental Science & Technology*

Includes: 31 pages, 11 Figures, 13 Tables, 6 Equations, and 8 Reaction Schemes

TABLE OF CONTENTS

Chemicals S4
Synthesis of 3-methyl-3,4-dihydro-2H-1,4-benzoxazine. S4
Synthesis of 2-amino-1-(2-furyl)ethanol S6
Calculation of second-order rate constants k_H and k_{OH} S8
Table S1: Hydraulic residence times for unit operations and distribution at the University of Iowa Drinking Water Treatment Plant S8
Analytical Methods S8
Table S2: HPLC mobile phase gradient S9
Table S3: Orbitrap Q-Exactive mobile phase gradient S9
Table S4: Average pH and temperature data for dichloroacetamide hydrolysis experiments under neutral conditions. S9
Table S5: Rate constants for dichloroacetamide safener hydrolysis at various concentrations of HCl or NaOH S10
Figure S1: Normalized concentration (C/C_0) of dichloroacetamide safeners in varied concentrations of HCl or NaOH S11
Figure S2: Half-lives [$t_{1/2} = \ln(2)/k_{obs}$] of each safener according to pH S12
Additional Discussion of Benoxacor Reactivity toward Hydroxide S12
Table S6: Benoxacor concentrations and system pH in samples collected from the UI DWTP softening basin, the Iowa River, and a laboratory tap S13
Table S7: Half-lives for benoxacor in softening basin samples when k_{obs} is calculated at various time points during experimentation to assess the influence of pH drift on hydrolysis rate. S13
Table S8: pH of unspiked environmental samples (i.e., systems without any added safener) measured at end of the four-day hydrolysis experiment. S13
Table S9: Benoxacor concentrations and system pH in either 5 mM borate (pH 8.4, 8.9) or carbonate (pH 10.7) buffers[1] S14
Calculation of Benoxacor Half-Life from k_{OH} S14
Calculation of Activity S15
Table S10: Mean activity coefficients of HCl and NaOH S15
Calculation of Ionic Strength S15
Table S11: Ionic strength and calculated half-lives for benoxacor based on measured rate constants for three hydrolysis systems S16
Figure S3: Normalized concentration plotted against time for benoxacor hydrolysis in 5 mM, pH 10.5 borate buffer held at 2, 21, or 35 °C S16
Table S12: Rate constants for benoxacor hydrolysis in pH 10.5, 5 mM sodium borate buffer S16
Calculation of Activation Energies S17
Table S13: Comparison of calculated activation energy for benoxacor to literature values for structurally-related organohalide herbicides S17
Scheme S1: Proposed products for AD-67 base-mediated hydrolysis S18
Figure S4: A) LC chromatogram and HRMS spectrum of benoxacor base-mediated hydrolysis product Benox-149 .... S18
Figure S5: During base-catalyzed hydrolysis of benoxacor (pH 10.5, 35° C), two transformation products were observed. S19
Scheme S2: Proposed mechanism for base-mediated benoxacor hydrolysis S19
Figure S6: LC chromatogram and HRMS spectrum of dichlormid base-mediated hydrolysis product, showing residual parent dichlormid S20

Figure S7: LC chromatogram and HRMS spectrum of dichlormid base-mediated hydrolysis product Dich 97 .... S21
Figure S8: Screen capture of Compound Discoverer software depicting peak areas for Dich-97 in an experimental sample and in an experimental sample augmented with addition of diallylamine standard S22
Scheme S3: Proposed mechanism for base-mediated dichlormid hydrolysis S22
Figure S9: LC chromatogram and HRMS spectrum of furilazole base-mediated hydrolysis product Furil-127 .... S24
Scheme S4: Proposed mechanism for base-mediated furilazole hydrolysis which yields Furil-127 and dichloroacetate. Ring opening is analogous to that proposed by Fife (1967)[9] S25
Figure S10: LC chromatogram and Orbitrap MS spectrum of AD-67 acid-mediated hydrolysis product AD-171 .... S26
Scheme S5: Proposed mechanism for formation of 153.9821 m/z peak as a result of ionization.......... S27
Scheme S6: Proposed mechanism for acid-mediated hydrolysis of AD-67. The highlighted compounds were observed via Orbitrap mass spectrometry. S27
Figure S11: LC chromatogram and HRMS spectrum of furilazole acid-mediated hydrolysis product Furil-237 .... S28
Scheme S7: Proposed mechanism for acid-mediated hydrolysis of furilazole S29
Scheme S8: Proposed mechanism for acid-mediated benoxacor hydrolysis S30

REAGENTS

Chemicals. Dichloroacetamide safeners used in bench experiments include: AD-67 (technical grade, donated by Nanjing Essence Fine-Chemical Co., CAS 71526-07-3, IUPAC 2,2-dichloro-1-(1-oxa-4-azaspiro[4.5]dec-4-yl)ethenone), benoxacor (99.4%, Sigma Aldrich, CAS 98730-04-2, IUPAC 2,2-dichloro-1-(3-methyl-2,3-dihydro-4H-1,4-benzoxazin-4-yl)ethenone), dichlormid (>97.0%, TCI America, CAS 37764-25-3, IUPAC 2,2-dichloro-N,N-di(prop-2-en-1-yl)acetamide), and furilazole (99.6%, Fluka, CAS 121776-33-8, IUPAC 2,2-dichloro-1-[5-(furan-2-yl)-2,2-dimethyl-1,3-oxazolidin-3-yl]ethan-1-one). The hydrolysis product diallylamine (99%, Sigma Aldrich, CAS 124-02-7, IUPAC N-prop-2-enylprop-2-en-1-amine) was purchased. Two hydrolysis products, 3-methyl-3,4-dihydro-2H-1,4-benzoxazine and 2-amino-1-(2-furyl)ethanol were synthesized as described below. For each safener, a 10 mM stock solution was prepared in HPLC-grade acetonitrile (Fisher Scientific). HPLC analysis used HPLC-grade acetonitrile and deionized water purified to 18 MΩ•cm. Optima grade acetonitrile and optima grade water were used for LC-MS analysis. Other chemicals used in experiments include hydrochloric acid, sodium hydroxide, and 5 mM potassium phosphate, sodium borate, or sodium bicarbonate buffers (made in lab using 18 MΩ•cm deionized water).

Synthesis of 3-methyl-3,4-dihydro-2H-1,4-benzoxazine. Synthesis was carried out in two stages: synthesis of 1-(2-nitrophenoxy)propan-2-one followed by ring closure to yield 3-methyl-3,4-dihydro-2H-1,4-benzoxazine:

1. Synthesis of 1-(2-nitrophenoxy)propan-2-one

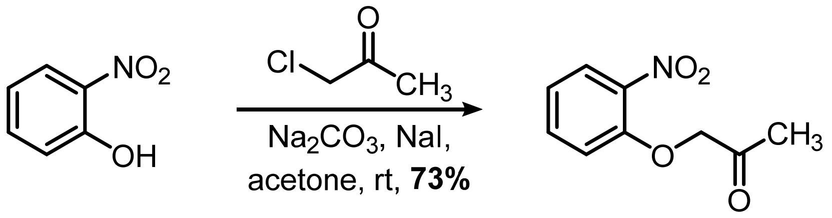

Note: Chloroacetone is a severe lachrymator, and this reaction must be carried out in an efficient fume hood.

To a flame-dried 100 mL flask under argon equipped with a magnetic stirring bar was added a solution of 2-nitrophenol (5.00 g, 35.9 mmol, 1.00 eq.) in 75 mL of acetone. Solid anhydrous sodium carbonate (7.62 g, 71.9 mmol, 2.00 eq.) was added, and the reaction mixture immediately turned orange. Solid sodium iodide (5.39 g, 35.9 mmol, 1.00 eq.) was then added followed by neat chloroacetone (4.29 mL, 53.9 mmol, 1.50 eq.), and the heterogeneous reaction mixture was stirred at room temperature for 16 hours. After this time, thin layer chromatography (2:1 hexanes / ethyl acetate, UV / anisaldehyde stain) showed complete consumption of the starting material ($R_f = 0.59$, stains faintly white) and clean formation of the product ($R_f = 0.22$, stains blue). The reaction mixture was filtered to remove the accumulated solids, and these were washed with several portions of dichloromethane. The solvent was removed under reduced pressure, and the crude product was partitioned between dichloromethane and 1 M aqueous hydrochloric acid solution.

After vigorous mixing, the layers were separated, and the aqueous phase was extracted with two additional portions of dichloromethane. The combined organic phases were dried over anhydrous sodium sulfate, the drying agent was removed by vacuum filtration, and the solvent was evaporated under reduced pressure. The crude product was purified by recrystallization (4:1 hexanes / ethyl acetate) to afford pure 1-(2-nitrophenoxy)propan-2-one (5.12 g, 73%) as a cream-colored solid.

Melting point: 66.7–67.4 °C

^{1}H NMR (400 MHz, $CDCl_3$): δ 7.90 (1H, dd, *J* = 8.0 Hz, 1.7 Hz), 7.54 (1H, td, *J* = 8.0 Hz, 1.7 Hz), 7.11 (1H, td, *J* = 8.0 Hz, 1.7 Hz), 6.95 (1H, d, *J* = 8.0 Hz), 4.63 (2H, s), 2.37 (3H, s).

^{13}C NMR (100 MHz, $CDCl_3$): 204.6, 151.1, 140.1, 134.5, 126.2, 121.7, 114.7, 73.9, 27.1.

IR: ṽ 3084, 2924, 1720, 1606, 1584, 1519, 1485, 1432, 1347, 1286, 1239, 1166, 1092, 1053, 970, 858, 820, 774, 742 cm^{-1}.

HRMS: *m/z* 218.0460 $[M+Na]^+$ (calculated for $C_9H_9NNaO_4$, 218.0424).

2. Synthesis of 3-methyl-3,4-dihydro-2H-1,4-benzoxazine

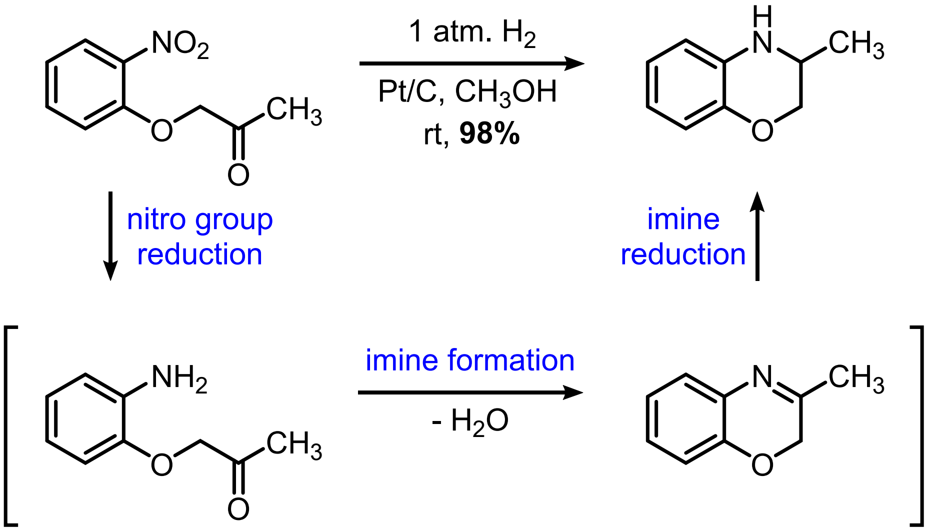

Note: Dry platinum on carbon is pyrophoric and should be handled under argon.

To a 500 mL flask under argon equipped with a magnetic stirring bar was added solid 1-(2-nitrophenoxy)propan-2-one (2.50 g, 12.6 mmol, 1.00 eq.), 5% platinum on carbon (1.00 g, 0.256 mmol, 0.02 eq.), and 150 mL of methanol. The flask was evacuated and put under an atmosphere of hydrogen gas using a balloon. The reaction mixture was stirred vigorously at room temperature for 16 hours, at which point thin layer chromatography (3:1 hexanes / ethyl acetate, UV /

anisaldehyde stain) showed complete consumption of the starting material (R_f = 0.15), and clean formation of the product (R_f = 0.40). The reaction mixture was filtered through a pad of Celite covered with a thin layer of silica gel to remove the platinum on carbon, and this pad was washed with dichloromethane. The solvent was removed under reduced pressure, and the crude product was purified by column chromatography on silica gel (4:1 hexanes / ethyl acetate) to give 3-methyl-3,4-dihydro-2H-1,4-benzoxazine (1.83 g, 98%) as a colorless oil.

^{1}H NMR (400 MHz, $CDCl_3$): δ 6.83 (1H, dd, J = 7.9 Hz, 1.5 Hz), 6.79 (1H, td, J = 7.5 Hz, 1.5 Hz), 6.68 (1H, td, J = 7.5 Hz, 1.5 Hz) 6.61 (1H, dd, J = 7.5 Hz, 1.5 Hz), 4.20 (1H, dd, J = 10.4 Hz, 2.8 Hz), 3.63 (1H, br s), 3.79 (1H, dd, J = 10.4 Hz, 8.1 Hz), 3.55 (1H, m), 1.19 (3H, d, J = 6.4 Hz).

^{13}C NMR (100 MHz, $CDCl_3$): δ 143.7, 133.6, 121.4, 118.8, 116.5, 115.5, 70.8, 45.2, 17.8.

IR: ṽ 3368, 3055, 2969, 2927, 2870, 1607, 1590, 1497, 1343, 1308, 1205, 1043, 739 cm^{-1}.

HRMS: *m/z* 150.0935 $[M+H]^+$ (calculated for $C_9H_{12}NO$, 150.0913).

Synthesis of 2-amino-1-(2-furyl)ethanol.[1]

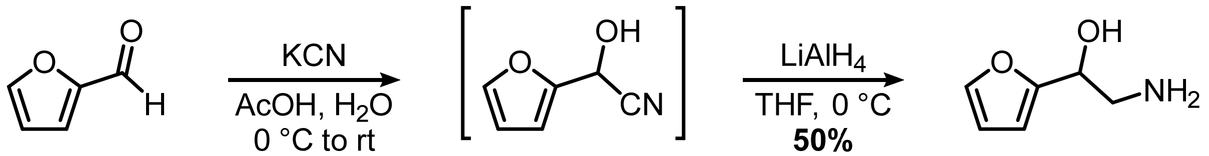

<u>Note:</u> Potassium cyanide is highly toxic and must be handled with care. Lithium aluminum hydride is pyrophoric and reacts exothermically with water. This chemical must be used under an inert atmosphere in a fume hood.

To a 100 mL flask under argon equipped with a magnetic stirring bar was added a solution of freshly distilled 2-furaldehyde (3.00 mL, 36.2 mmol, 1.00 eq.) in 18 mL of acetic acid (AcOH). This colorless solution was cooled to 0 °C in an ice bath, and a solution of potassium cyanide (5.66 g, 86.9 mmol, 2.40 eq.) in 18 mL of water was added dropwise over 15 minutes. After addition was complete, the resulting yellow solution was allowed to warm to room temperature and stirred for 16 hours. The reaction mixture was diluted with water and extracted three times with diethyl ether before the combined organic layers were dried over anhydrous Na_2SO_4. Thin layer chromatography (3:1 hexanes / ethyl acetate, UV / anisaldehyde stain) showed complete consumption of the aldehyde (R_f = 0.39) and formation of the cyanohydrin (R_f = 0.30, stains blue). The drying agent was removed by vacuum filtration, and the solvent was evaporated under reduced pressure to afford the crude cyanohydrin (5.03 g) as a yellow oil that was used without purification. ^{1}H-NMR analysis showed that this crude material contained ~16% acetic acid (by mass) and so a larger excess of lithium aluminum hydride was used in the subsequent reduction step.

To a flame-dried 500 mL flask under argon equipped with a large magnetic stirring bar was added 120 mL of anhydrous tetrahydrofuran (THF). The flask was cooled to 0 °C in an ice bath, and a

3.5 M solution of lithium aluminum hydride in toluene (32.8 mL, 114.8 mmol, 3.17 eq.) was added through a septum using a syringe. A solution of the crude cyanohydrin (5.03 g) in 60 mL of anhydrous THF was then added dropwise over 20 minutes. After the addition was complete, the resulting dark yellow solution was stirred at 0 °C for 2 hours. The reaction was then carefully quenched by the slow, dropwise addition of 4.37 mL of water, followed by 4.37 mL of 15% aq. NaOH solution, and finally 13 mL of water. Solid anhydrous Na_2SO_4 (15 g) was then added, and the mixture was stirred vigorously at room temperature for 30 minutes. The heterogeneous reaction mixture was filtered through Celite, and the powdery solid (aluminum salts mixed with drying agent) was washed several times with diethyl ether. Thin layer chromatography (1:1 hexanes / ethyl acetate, UV / anisaldehyde stain) of the filtrate showed complete consumption of the cyanohydrin (R_f = 0.57), a byproduct of R_f = 0.45 (UV-active), and the desired amino alcohol on the baseline (R_f = 0, stains black). The solvent was evaporated under reduced pressure to give the crude amino alcohol (4.29 g) as a light yellow solid. This material was recrystallized from ~40 mL of acetonitrile to afford the pure amino alcohol as a white crystalline solid (2.2978 g, 50% over two steps).

Melting point: 87.2–89.2 °C

^{1}H NMR (400 MHz, $CDCl_3$): δ 7.35 (1H, d, *J* = 1.6 Hz), 6.31 (1H, dd, *J* = 3.2 Hz, 1.6 Hz), 6.23 (1H, d, *J* = 3.2 Hz), 4.61 (1H, t, *J* = 5.8 Hz), 2.98 (2H, d, *J* = 5.8 Hz), 2.80–2.00 (3H, br s).

^{13}C NMR (100 MHz, $CDCl_3$): 155.6, 142.1, 110.3, 106.4, 68.2, 46.1.

IR (solid ATR)**:** ṽ 3347, 3277, 3104, 2927, 2819, 2738, 1600, 1496, 1465, 1436, 1365, 1296, 1233, 1203, 1162, 1138, 1097, 1064, 1021, 965, 938, 879, 935, 818, 787, 746, 653 cm^{-1}.

METHOD DETAILS

Calculation of second-order rate constants k_H and k_{OH}. Second order rate coefficients were calculated from experimentally determined pseudo-first-order rate constants assuming exponential decay in safener concentration over time in acidic, basic and neutral pH systems (Eq. S1 and S2). This approximation is appropriate given the sufficient molar excess of $[H^+]$, $[OH^-]$ and $[H_2O]$ in acidic, basic and neutral pH experimental systems, respectively.

$$k_H = \frac{k_{obs}}{[H^+]} \quad \textbf{(Eq. S1)}$$

$$k_{OH} = \frac{k_{obs}}{[OH^-]} \quad \textbf{(Eq. S2)}$$

Table S1: Hydraulic residence times for unit operations and distribution at the University of Iowa Drinking Water Treatment Plant. Ranges based on minimum expected flow (2.0 MGD) and maximum expected flow (4.25 MGD), with distribution time to the most distant point of use location.

Operation	*Residence Time (h)*
Flocculation and Sedimentation	2.7 – 5.7
Lime-Soda Softening	1.5 – 3.2
Filtration	1.1 – 2.3
Distribution	0 – 1.0
Total	5.3 – 12.2

Analytical Methods: Dichloroacetamide samples were quantified using an Agilent 1260 HPLC with Diode Array Detection (HPLC-DAD) with OpenLab ChemStation software (Waldbronn, Germany). HPLC methods generally follow those developed by Kral *et al.* using an Agilent Zorbax Eclipse XDB-C18 column (4.6 × 150 mm, 3.5 µm), acetonitrile and water gradated eluent (Table S2) at a flow rate of 1 mL/min, and 25 µL injection volumes.[2] Analytes were monitored at 220 nm, and concentrations of parent compounds were quantified using a five-point calibration curve. For purposes of detecting analytes in endogenous environmental samples, the limit of detection (LOD) was determined as the amount of analyte that produced a signal greater than three times the background signal. Because the lowest calibration curve concentration for all safeners was 1 µmol/L, the sample LOD is 3 µmol/L.

For structure identification of transformation products, samples were analyzed using an Orbitrap Q-Exactive mass spectrometer (Thermo Fisher Scientific, Bremen, Germany) equipped with a heated ESI source. Analysis used the same Agilent Zorbax Eclipse XDB-C18 column and a similar eluent method (Table S3) to that used in the HPLC analysis. The injection volume was 10 µL. Analysis was carried out in both positive (ESI+) and negative (ESI-) electrospray ionization modes. The operating parameters were as follows: sheath gas, auxiliary gas, sweep gas, 58, 16, 3 (arbitrary units) respectively; spray voltage, 3.5 kV; temperature of ion transfer capillary, 281 °C; S-lens RF level, 80. Stepped collision energies for fragmentation were 20, 30, and 40 V.

Mass calibration of the instrument was performed at least every 7 days using the calibration solutions.

Data were acquired using Full MS/dd-MS^2 (Top N) mode. With this mode, all ions present in the sample were collected in the quadrupole during the first scan event (full MS scan). The second scan event (dd-MS^2) was performed to obtain all fragmented ions from the top five most abundant parent ions identified during the first scan event. Data were analyzed using XCalibur (Thermo Fisher Scientific, version 4.2.47) and Compound Discoverer (Thermo Fisher Scientific, version 3.1.0.305) software. Compound Discoverer used a processing workflow built for untargeted analysis of environmental samples. The workflow performed retention time alignment, unknown compound detection, and compound grouping across all samples.

Table S2: HPLC mobile phase gradient

Time (min)	% Acetonitrile	% Deionized Water
0	25	75
11	80	20
12	25	75
15	25	75

Table S3: Orbitrap Q-Exactive mobile phase gradient

Time (min)	% Acetonitrile	% Deionized Water
0	25	75
2	25	75
13	90	10
14	25	75
22	25	75

SUPPORTING RESULTS

Table S4: Average pH and temperature data for dichloroacetamide hydrolysis experiments under neutral conditions.

	AD-67			Benoxacor			Dichlormid			Furilazole		
Time (d)	C/C_0	pH	Temperature (°C)	C/C_0	pH	Temperature (°C)	C/C_0	pH	Temperature (°C)	C/C_0	pH	Temperature (°C)
0	1.000	7.1	20.2	1.000	7.0	19.9	1.000	7.0	20.0	1.000	7.0	20.1
7	0.987	7.1	21.7	0.955	7.1	21.9	1.007	7.0	21.0	0.996	7.0	21.3
14	0.978	7.1	21.6	0.893	7.0	21.3	1.004	7.0	21.2	0.999	7.0	21.0
21	0.977	7.0	21.8	0.814	7.0	22.0	1.001	7.0	21.8	0.997	7.0	21.4
28	0.971	6.9	22.0	0.740	7.0	21.8	0.998	6.9	21.8	0.996	7.0	21.5
42	0.968	6.9	22.0	0.568	7.0	21.8	0.987	6.9	21.8	0.994	6.9	22.0

Table S5: Rate constants for dichloroacetamide safener hydrolysis at various concentrations of HCl or NaOH. Values were calculated based on H^+ and OH^- concentrations and activities.

Safener	$[H^+]$	$[OH^-]$	k_H (M^{-1} h^{-1})	k_N (h^{-1})	k_{OH} (M^{-1} h^{-1})	Activity H^+	Activity OH^-	Activity-based k_H (M^{-1} h^{-1})	Activity-based k_N (h^{-1})	Activity-based k_{OH} (M^{-1} h^{-1})
AD-67	2		0.61			2.02		0.60		
AD-67	2		0.54			2.02		0.54		
AD-67	1.5		0.40			1.33		0.45		
AD-67	1		0.30			0.81		0.37		
AD-67		1.5			0.47		1.03			0.68
AD-67		1			0.28		0.67			0.42
AD-67		0.5			0.14		0.34			0.20
**AD-67 Average**			**0.46**		**0.30**			**0.49**		**0.43**
**AD-67 Std. Dev.**			**0.14**		**0.17**			**0.10**		**0.24**
Benoxacor	2.5		3.32×10^{-3}			2.87		2.89×10^{-3}		
Benoxacor	2		1.15×10^{-3}			2.02		1.14×10^{-3}		
Benoxacor	2		3.84×10^{-3}			2.02		3.80×10^{-3}		
Benoxacor		0.01			785.40		0.0090			870.73
Benoxacor		0.008			622.88		0.0077			649.91
Benoxacor		0.006			460.50		0.0058			480.49
Benoxacor		0.004			295.00		0.0037			315.12
Benoxacor	-	-		4.85×10^{-3}					4.85×10^{-3}	
Benoxacor	-	-		5.44×10^{-4}					5.44×10^{-4}	
Benoxacor	-	-		5.48×10^{-4}					5.48×10^{-4}	
**Benoxacor Average**			**2.77×10^{-3}**	**5.25×10^{-4}**	**540.94**			**2.61×10^{-3}**	**5.25×10^{-4}**	**579.06**
**Benoxacor Std. Dev.**			**1.43×10^{-3}**	**3.54×10^{-5}**	**210.90**			**1.36×10^{-3}**	**3.54×10^{-5}**	**237.68**
Dichlormid		1			5.13		0.67			7.61
Dichlormid		0.5			2.59		0.34			3.79
Dichlormid		0.5			2.61		0.34			3.81
Dichlormid		0.2			1.40		0.17			1.61
**Dichlormid Average**					**2.93**					**4.20**
**Dichlormid Std. Dev.**					**1.57**					**2.44**
Furilazole	2		2.44×10^{-2}			2.02		2.42×10^{-2}		
Furilazole	2		3.28×10^{-2}			2.02		3.25×10^{-2}		
Furilazole	1.5		2.79×10^{-2}			1.33		3.15×10^{-2}		
Furilazole	1		4.07×10^{-2}			0.81		5.02×10^{-2}		
Furilazole		2			4.52		2.05			4.41
Furilazole		1			4.86		0.67			7.21
Furilazole		0.5			3.71		0.34			5.42
Furilazole		0.5			0.88		0.34			1.28
**Furilazole Average**			**3.14×10^{-2}**		**3.49**			**3.46×10^{-2}**		**4.58**
**Furilazole Std. Dev.**			**7.09×10^{-3}**		**1.80**			**1.11×10^{-2}**		**2.48**

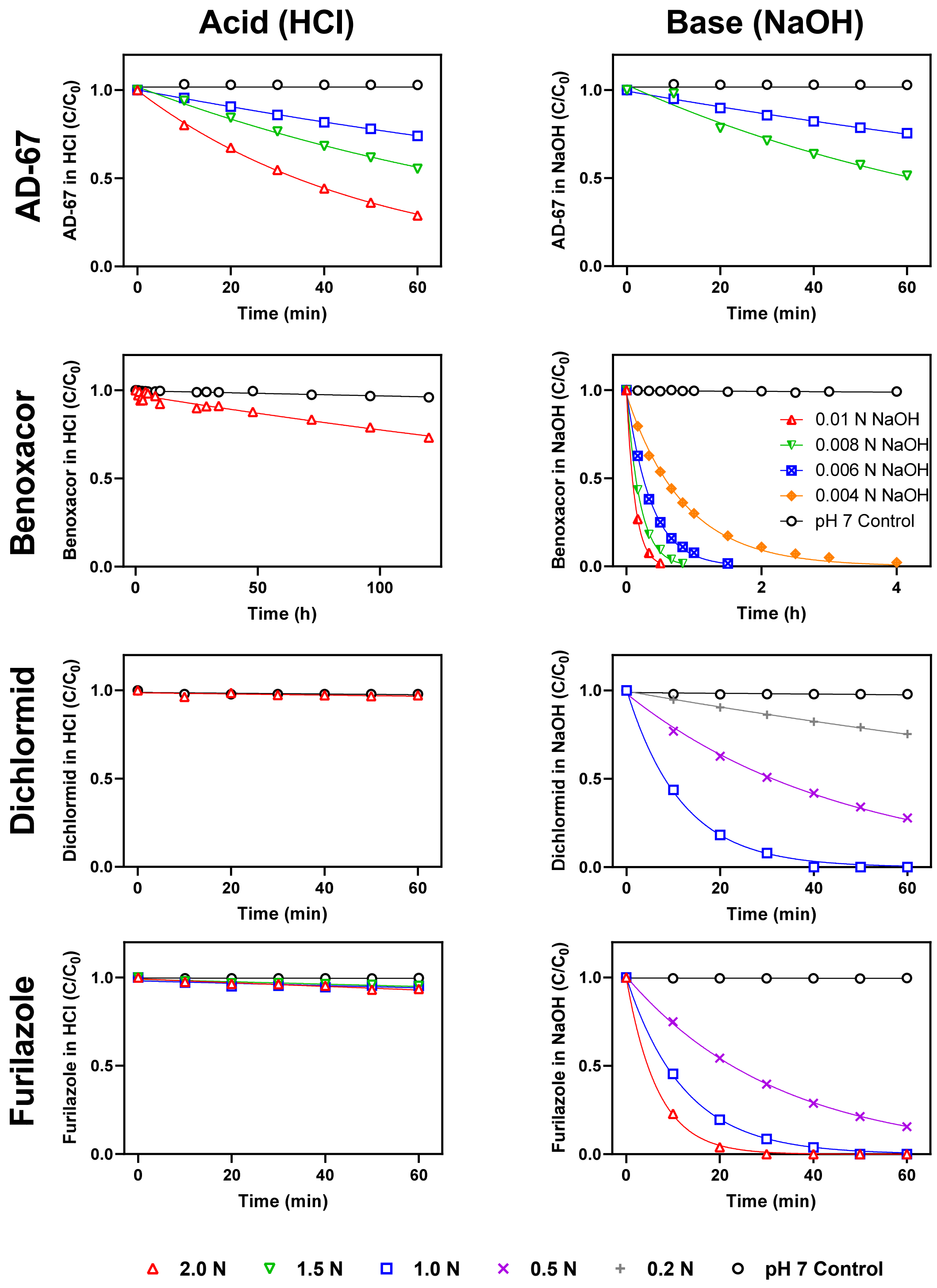

Figure S1: Normalized concentration (C/C_0) of dichloroacetamide safeners in varied concentrations of HCl or NaOH, with pH 7 buffered controls for comparison. Solid lines represent regression based on pseudo-first-order transformation kinetics. Experiments were conducted at ambient temperature (22 ± 2 °C). HCl and NaOH concentrations shown here were not adjusted for activity. The legend at the bottom of the figure applies to all frames except base-mediated hydrolysis of benoxacor.

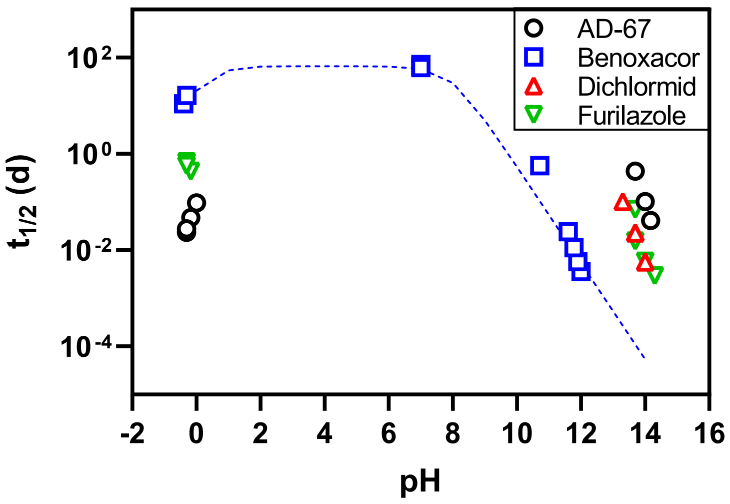

Figure S2: Half-lives [$t_{1/2} = \ln(2)/k_{obs}$] of each safener as a function of pH for systems where k_{obs} is statistically greater than 0. Benoxacor hydrolysis was modeled across pH 0 to 14 using Equation 1 in the main text to estimate half-lives at pH values not explored experimentally.

Additional Discussion of Benoxacor Reactivity toward Hydroxide. It is noted that the second-order rate constant for benoxacor base-mediated hydrolysis (k_{OH}) is at least two orders of magnitude larger than those measured for the other safeners. At least three possible rationales could account for this difference:

1) The environment surrounding the amide in benoxacor is sterically hindered due to the methyl group and the aromatic ring. This could result in a conformational change whereby the amide rotates out of planarity, leading to more facile hydrolysis due to decreased resonance stabilization.
2) Because the lone pair on N is shared with the aromatic ring in an extended pi system, benoxacor's amide has decreased resonance stabilization compared to the other safeners.
3) A second potential hydrolysis mechanism exists for sterically-hindered amides that contain electron-withdrawing groups in the alpha position. The mechanism involves an initial deprotonation at the α-positon and elimination resulting in formation of a ketene.[10] Although prior research has identified a mechanism similar to that which we propose in Scheme 1 for structurally-related chloroacetamide herbicides, the protonation-elimination mechanism could be more important for benoxacor because the aniline anion is a better leaving group compared to a standard amine due to resonance.

Table S6: Benoxacor concentrations and system pH in samples collected from the UI DWTP softening basin, the Iowa River, and a laboratory tap.

	Softening Basin		Iowa River		Tap Water	
**Time (d)**	**Benoxacor (μM)**	**pH**	**Benoxacor (μM)**	**pH**	**Benoxacor (μM)**	**pH**
0	12.31	10.71	12.29	8.39	12.40	8.91
0.167	11.54	10.60	12.23	8.49	12.27	8.81
0.33	10.99	10.57	12.21	8.58	12.25	8.72
1	9.57	10.46	12.08	8.56	12.18	8.62
2	8.19	10.40	11.91	8.45	12.14	8.57
3	7.28	10.27	11.75	8.40	12.07	8.53
4	6.65	10.15	11.58	8.36	11.99	8.48

Table S7: Half-lives for benoxacor in softening basin samples when k_{obs} is calculated at various time points during experimentation to assess the influence of pH drift on hydrolysis rate.

Time (d)	pH drift from t_0 (pH units)	k_{obs} (d^{-1})	Half-life (d)
0.33	0.14	0.341	2.0
1	0.25	0.246	2.8
4	0.56	0.161	4.3

Table S8: pH of unspiked environmental samples (i.e., systems without any added safener) measured at end of the four-day hydrolysis experiment.

System	Average pH
Softening basin	10.65
Iowa River water	8.34
Laboratory tap water	8.91

Table S9: Benoxacor concentrations and system pH in either 5 mM borate (pH 8.4, 8.9) or carbonate (pH 10.7) buffers[1]

	pH 8.4 Buffer		pH 8.9 Buffer		pH 10.7 Buffer	
**Time (d)**	**Benoxacor (μM)**	**pH**	**Benoxacor (μM)**	**pH**	**Benoxacor (μM)**	**pH**
0	12.21	8.38	12.32	8.91	12.62	10.67
0.167	12.09	8.41	12.17	8.92	9.91	10.67
0.33	12.07	8.41	12.11	8.93	7.88	10.66
1	12.06	8.44	12.08	8.93	6.29	10.65
2	11.95	8.43	11.86	8.94	3.84	10.67
3	11.79	8.43	11.49	8.95	1.02	10.64
4	11.67	8.43	11.22	8.93	0.34	10.65

[1]Eight hours after spiking with benoxacor, there was no significant difference between the pH of the tap water samples and that of the river water samples (Table S7, p = 0.09); as such, there was no significant difference in benoxacor hydrolysis rate constants for each system (p = 0.12). The calculated half-lives were 60 (± 30) days in tap water and 104 (± 16) days in river water. When the same experiment was conducted in a 5 mM sodium borate buffer that maintained a stable pH of 8.9 or 8.4 (Table S9), hydrolysis rates increased significantly compared to spiked environmental samples (p = 0.02 and p = 0.002, respectively). Benoxacor half-lives were 22.5 (± 1.9) and 53 (± 5) days in pH 8.9 and pH 8.4 buffered systems, respectively.

Calculation of Benoxacor Half-Life from k_{OH}: Based on k_{OH} values shown in Table S5, an estimated half-life was calculated for benoxacor at pH 7. Including error (as standard deviation), the k_{OH} for benoxacor ranges from 330 to 750 M^{-1} h^{-1}. At pH 10.7, $[OH^-] = 5.012 \times 10^{-4}$ M. The range of estimated hydrolysis rates at pH 10.7 is:

$$k = k_{OH} \times [OH^-] \qquad \textbf{(Eq. S3)}$$

Lower limit k: $(330\ M^{-1}\ h^{-1})(5.012 \times 10^{-4}\ M) = 0.165\ h^{-1}$

Upper limit k: $(751\ M^{-1}\ h^{-1})(5.012 \times 10^{-4}\ M) = 0.376\ h^{-1}$

Therefore, assuming pseudo-first-order decay [$t_{1/2} = \ln(2)/k$], the estimated half-life is 1.8 – 4.2 hours.

Calculation of Activity: Activity is a measure of the effective concentration of a species under non-ideal conditions where solute-solute interactions are non-negligible, determining the real chemical potential for a solution.[3] Because our experiments were conducted at elevated concentrations of $[H^+]$ and $[OH^-]$ (as well as their acid and base counterions Cl^- and Na^+, respectively), the possibility exists for non-ideal effects resulting from electrostatic forces between ions. Therefore, ion activity was determined according to Eq. S8:

$$\{i\} = \gamma_i [m_i] \qquad \textbf{(Eq. S4)}$$

Where {i} is the ion activity, γ_i is the measured or calculated activity coefficient, and $[m_i]$ is the ion molar concentration in water.[3]

HCl and NaOH concentrations used in experiments exceeded the limits of the Debye-Hückel (0.1 M) and Davies (0.5 M) equations for calculating γ_i in dilute solutions. As such, activity coefficients were obtained from the literature where they had been empirically measured and H^+ and OH^- activities were calculated.[4]

Table S10: Mean activity coefficients of HCl and NaOH, adapted from Hamer *et al.*[4]

[HCl]	γ	[NaOH]	γ
2.5	1.148	2	1.023
2	1.009	1.5	0.68775
1.5	0.887	1	0.674
1	0.811	0.5	0.685
		0.2	0.87
		0.01	0.902
		0.008	0.9584
		0.006	0.9584
		0.004	0.93615

Calculation of Ionic Strength: Ionic strength of strong acid/strong base and buffered solutions was calculated according to the Eq S5:

$$I = \frac{1}{2}\sum_i (z_i)^2 C_i \qquad \textbf{(Eq. S5)}$$

Where z_i is the charge number on each ion in the solution and C_i is the ion concentration [M].[3]

The ionic strength equation was used in combination with the Henderson-Hasselbach equation (for speciation of weak acids and weak bases) to calculate ionic strengths for the experimental systems in Table S11:

Table S11: Ionic strength and calculated half-lives for benoxacor based on measured rate constants for three hydrolysis systems

System	I (mM)	Calculated half-life (h)
5 mM sodium carbonate buffer, pH 10.7	10.28	13.8
5 mM sodium borate buffer, pH 8.9	7.73	537 (22.5 days)
5 mM sodium borate buffer, pH 8.4	6.15	1265 (52.9 days)
0.006 N NaOH	6	0.25
0.008 N NaOH	8	0.14

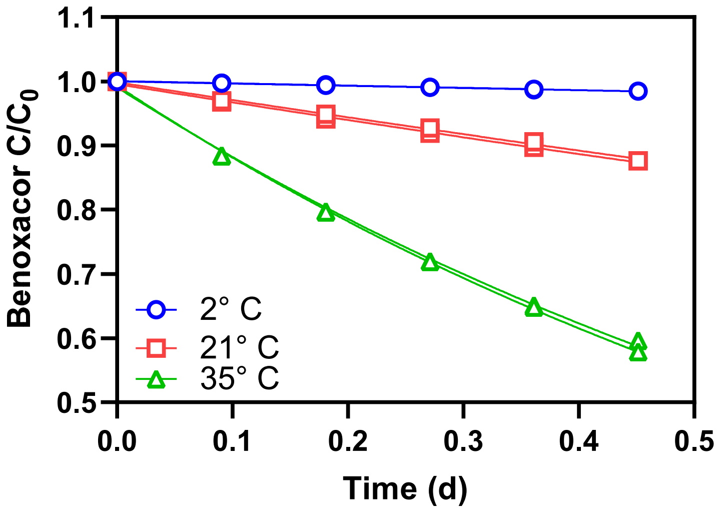

Figure S3: Normalized concentration plotted against time for benoxacor hydrolysis in 5 mM, pH 10.5 borate buffer held at 2, 21, or 35 °C. Solid lines represent fitted exponential curves based on a pseudo-first order model. Experiments were conducted in duplicate, with all data points displayed on the plot.

Table S12: Rate constants for benoxacor hydrolysis in pH 10.5, 5 mM sodium borate buffer. These rate constants were plotted against inverse temperature to determine the activation energy for benoxacor at the specified pH.

Temperature (°C)	Pseudo-first-order rate constant (± standard deviation) (d^{-1})
2	0.036 ± 0.001
21	0.28 ± 0.02
35	1.17 ± 0.04

Calculation of Activation Energies. The Arrhenius model[5] was used to assess temperature dependence by estimating activation energies from the pseudo-first-order rate constants at three temperatures representative of those typically encountered in surface waters (2° C, 21°C, and 35°C, Figure S3).

$$E_a = \frac{-R\ln(\frac{k_1}{k_2})}{\frac{1}{T_2}-\frac{1}{T_1}} \qquad \textbf{(Eq. S6)}$$

Where E_a is the activation energy (kJ mol^{-1}), R is the universal gas constant (kJ mol^{-1} K^{-1}), k_1 and k_2 are rate constants (h^{-1}) at temperature T_1 and T_2 (K). The activation energy was determined by relating the natural log of the pseudo-first-order rate constant at three temperatures to the inverse temperature (i.e., the slope × 8.314 × 10^{-3} kJ mol^{-1} K^{-1} in Equation S6). The activation energy was determined as 75 ± 2 kJ mol^{-1}.

Table S13: Comparison of calculated activation energy for benoxacor to literature values for structurally-related organohalide herbicides

**Compound**	**Temperature range (°C)**	**Hydrolysis pH**	**Activation Energy (kJ mol^{-1})**	**Source**
Benoxacor	2 – 35	10.5	75 ± 2	This study
Atrazine	20 – 30	2	124.4	2018[6]
		4	42.5	
		12	95.4	
Acetochlor	20 – 30	12	106.4	Masbou, 2018[6]
Alachlor	20 – 30	12	91.6	Masbou, 2018[6]
Butachlor	20 – 30	12	73.4	Masbou, 2018[6]
Chlorpyrifos	15 – 35	4.7	95.4	Meikle, 1978[7]
		6.9	79.5	
		8.1	91.2	

Base-Mediated Hydrolysis

AD-67: For base-mediated hydrolysis, dichloroacetate was detected, suggesting a base-mediated acyl cleavage mechanism that would also yield 1-Oxa-4-azaspiro[4.5]decane, but that product was not observed.

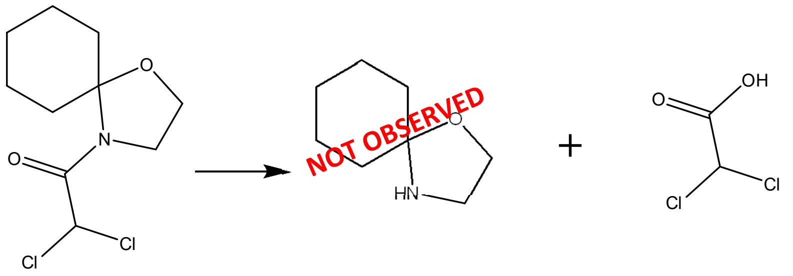

Chemical Formula: $C_{10}H_{15}Cl_2NO_2$ Exact Mass: 251.04798

Chemical Formula: $C_8H_{15}NO$ Exact Mass: 141.11536

Chemical Formula: $C_2H_2Cl_2O_2$ Exact Mass: 127.94318

Scheme S1: Proposed products for AD-67 base-mediated hydrolysis. Dichloroacetate was detected, suggesting the presence of 1-Oxa-4-azaspiro[4.5]decane, but no other products were observed by Orbitrap LC-MS/MS.

Benoxacor: 3-methyl-3,4-dihydro-2H-1,4-benzoxazine was identified in a base-mediated system with Level 1 confidence.

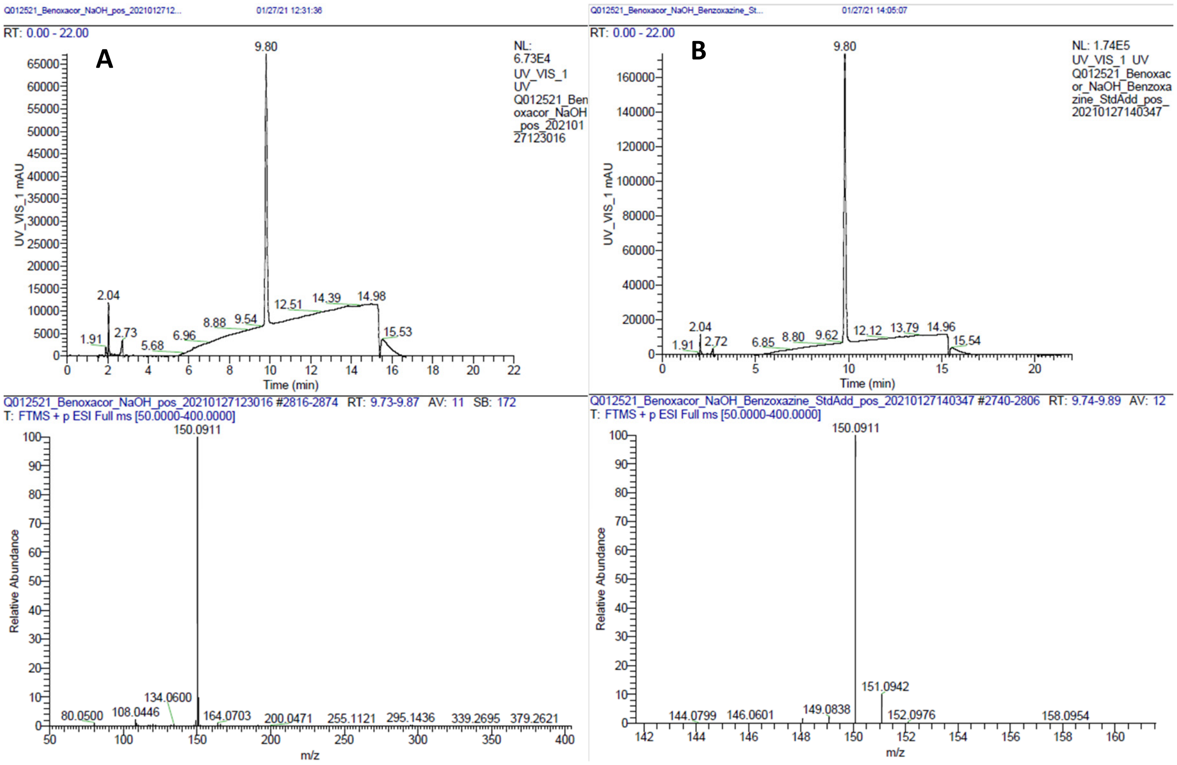

Figure S4: A) LC chromatogram and HRMS spectrum of benoxacor base-mediated hydrolysis product Benox-149, and B) LC chromatogram and HRMS spectrum for the same benoxacor

base-mediated hydrolysis sample, with a small amount of 3-methyl-3,4-dihydro-2H-1,4-benzoxazine standard added. The addition resulted in a peak area increase for the 9.8 minute peak, and the m/z ratio remained consistent, demonstrating Level 1 confidence[8] that the product is 3-methyl-3,4-dihydro-2H-1,4-benzoxazine.

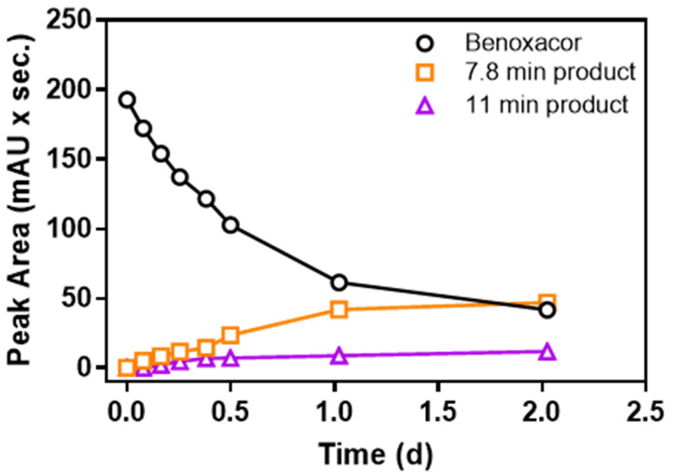

Figure S5: During base-catalyzed hydrolysis of benoxacor (pH 10.5, 35° C), two transformation products were observed. The 7.8 minute product was identified as 3-methyl-3,4-dihydro-2H-1,4-benzoxazine. We suspect the 11-minute product results from 3-methyl-3,4-dihydro-2H-1,4-benzoxazine oxidation and was not observed by Orbitrap MS nor gas chromatography.

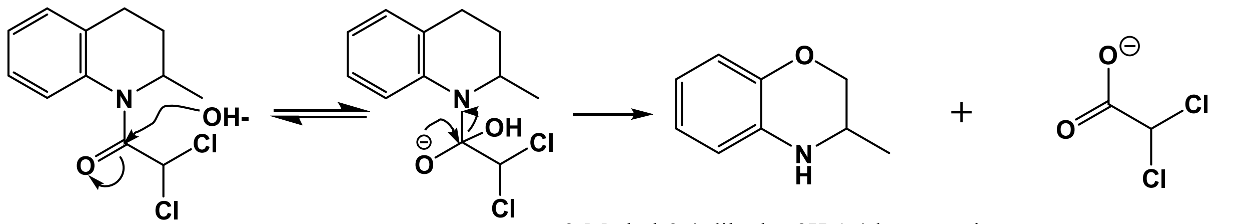

Benoxacor
Exact Mass: 259.0167

3-Methyl-3,4-dihydro-2H-1,4-benzoxazine
Exact Mass: 149.0841

Dichloroacetate
Exact Mass: 126.9359

Scheme S2: Proposed mechanism for base-mediated benoxacor hydrolysis which yields 3-methyl-3,4-dihydro-2H-1,4-benzoxazine and dichloroacetate.

Dichlormid: Base-catalyzed hydrolysis of dichlormid yields diallylamine (Dich-97; verified with Level 1 confidence)[8] and dichloroacetate. We propose that dichlormid undergoes the same base-mediated hydrolysis mechanism as benoxacor.

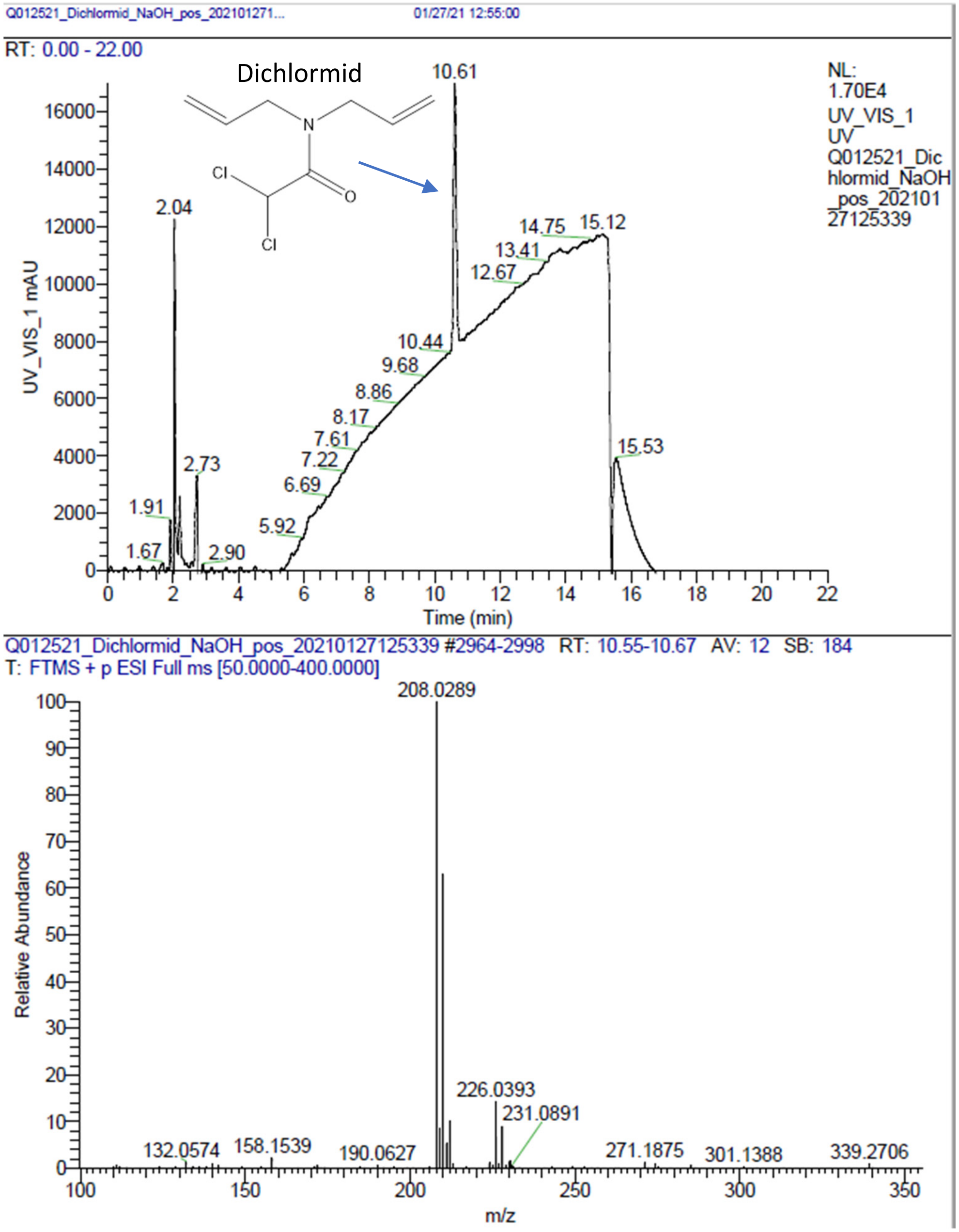

Figure S6: LC chromatogram and HRMS spectrum of dichlormid base-mediated hydrolysis product, showing residual parent dichlormid

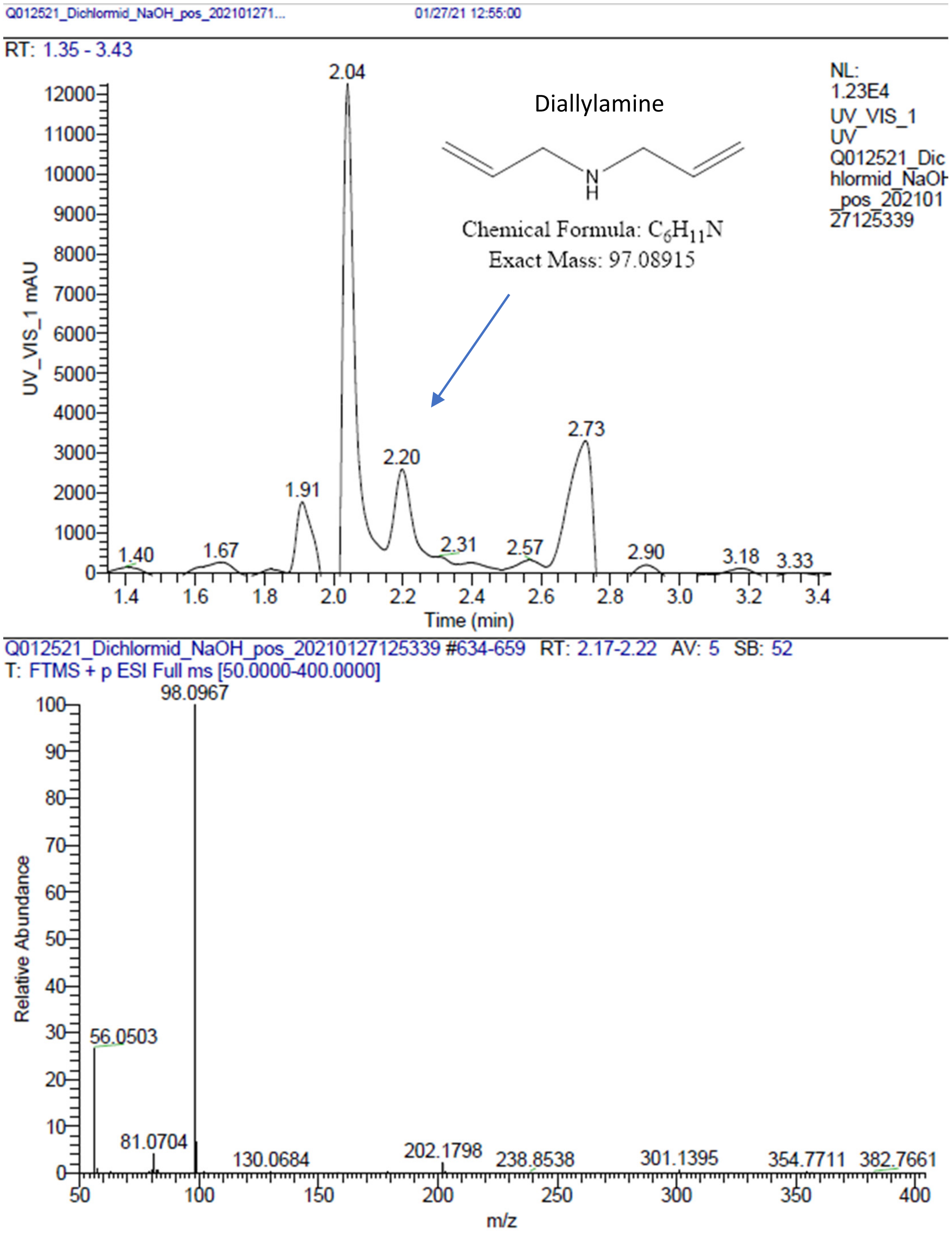

Figure S7: LC chromatogram and HRMS spectrum of dichlormid base-mediated hydrolysis product Dich 97 (also known as diallylamine) in ESI-positive mode. Dichloroacetate was observed in negative mode.

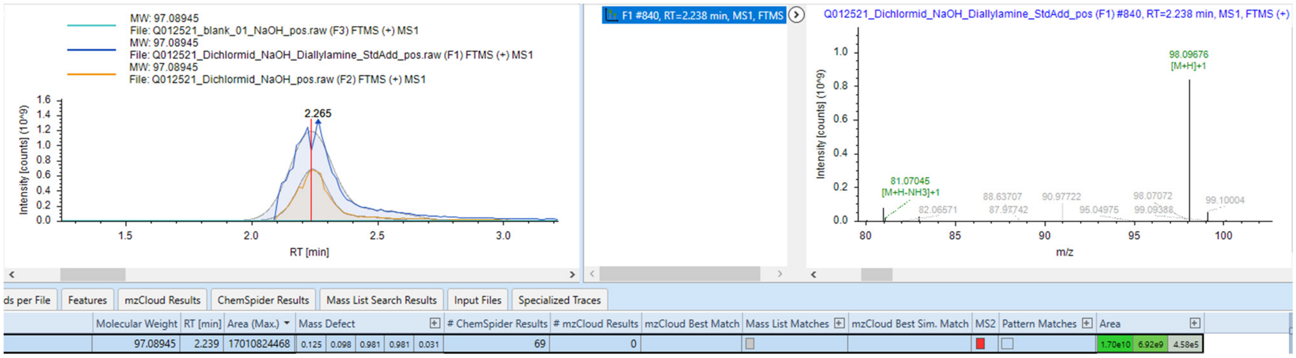

Figure S8: Screen capture of Compound Discoverer software depicting peak areas for Dich-97 in an experimental sample and in an experimental sample augmented with addition of diallylamine standard. The peaks overlap and produce the same MS spectra, demonstrating Level 1 confidence that the product is diallylamine. MS^2 data was not available for diallylamine.

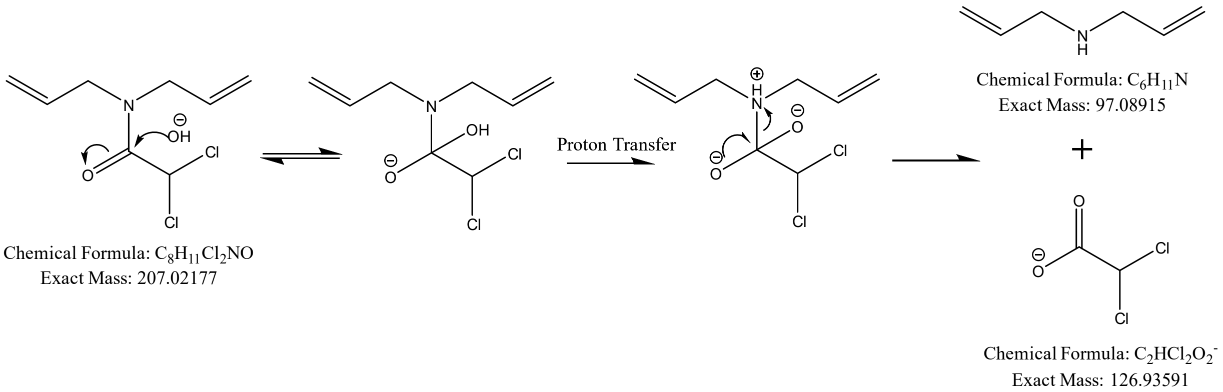

Scheme S3: Proposed mechanism for base-mediated dichlormid hydrolysis which yields diallylamine and dichloroacetate.

<u>Furilazole:</u> Base-mediated hydrolysis of furilazole yielded Furil-127, which was identified as 2-amino-1-(2-furyl)ethanol with Level 1 confidence using a synthesized reference standard. Dichloroacetate was also observed as a product of furilazole base-mediated hydrolysis.

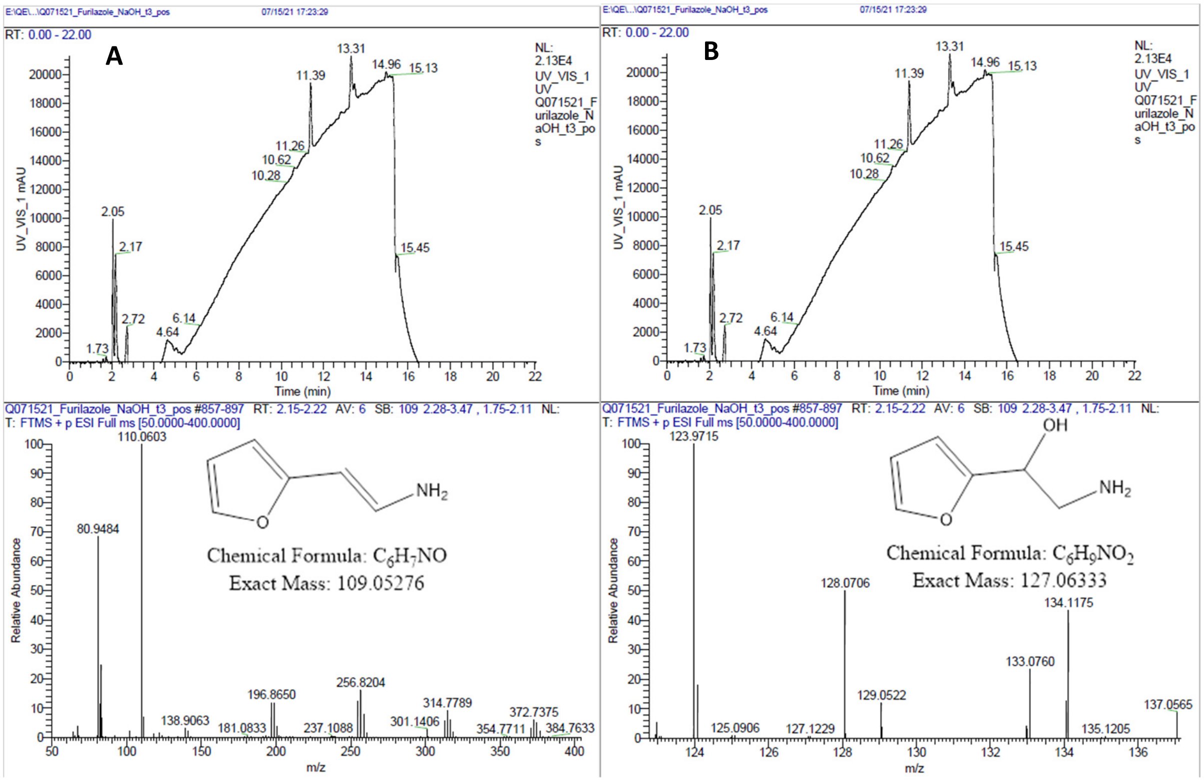

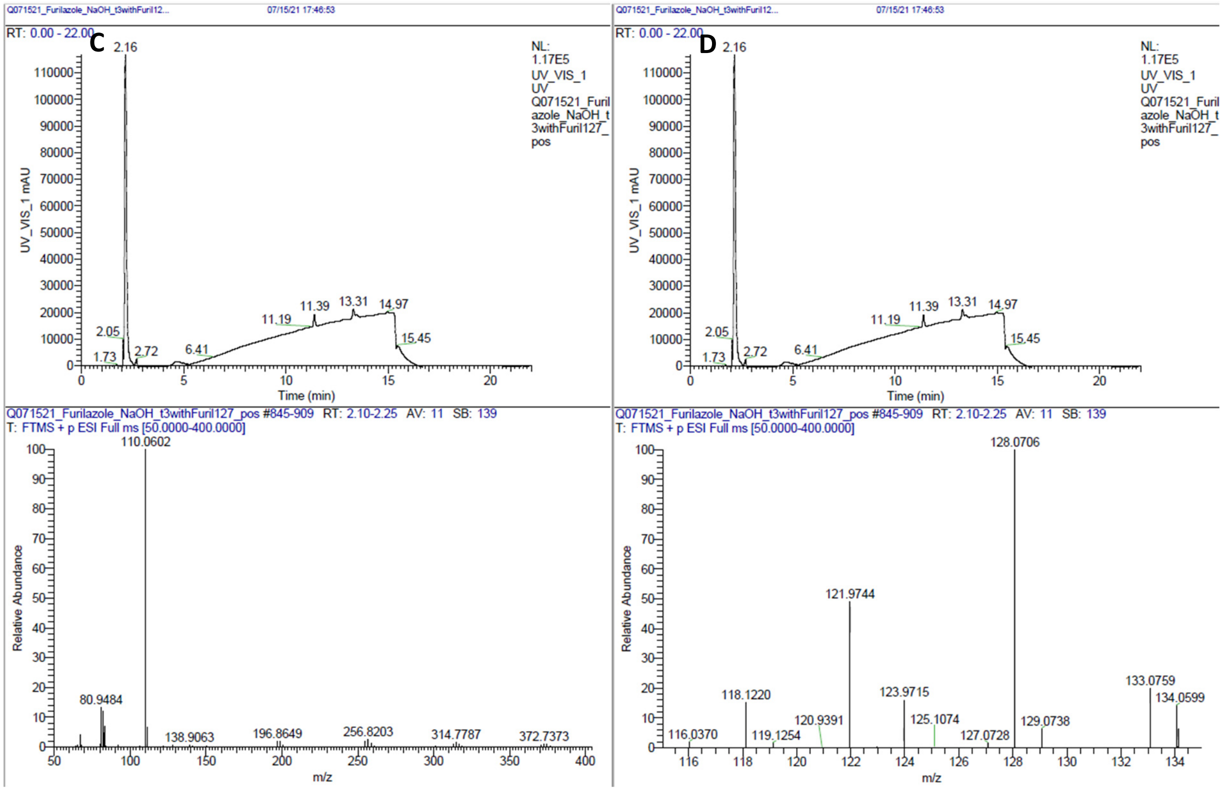

Figure S9: LC chromatogram and HRMS spectrum of furilazole base-mediated hydrolysis product Furil-127. A) shows the full LC-MS chromatogram with the m/z spectra of the peak of interest at 2.17 minutes. B) shows the LC-MS chromatogram with m/z spectra zoomed in on the mass of interest at 128.0708 mass units. C) shows the full LC-MS chromatogram and m/z spectra at 2.16 minutes for the same base-mediated furilazole hydrolysis sample, with a small amount of 2-amino-1-(2-furyl)ethanol (i.e., Furil-127) added. D) shows the zoomed-in LC-MS spectra and m/z spectra for the sample with 2-amino-1-(2-furyl)ethanol added. Although the corresponding m/z peak is small for the Furil-127 $[M+H]^+$ (1.3×10^6 peak area units, which is 2-3 orders of magnitude smaller than most other identified safener products), Compound Discoverer software identifies the much larger peak (peak area 1×10^9) at m/z = 110 as an MS^2 fragment of the 127 product.

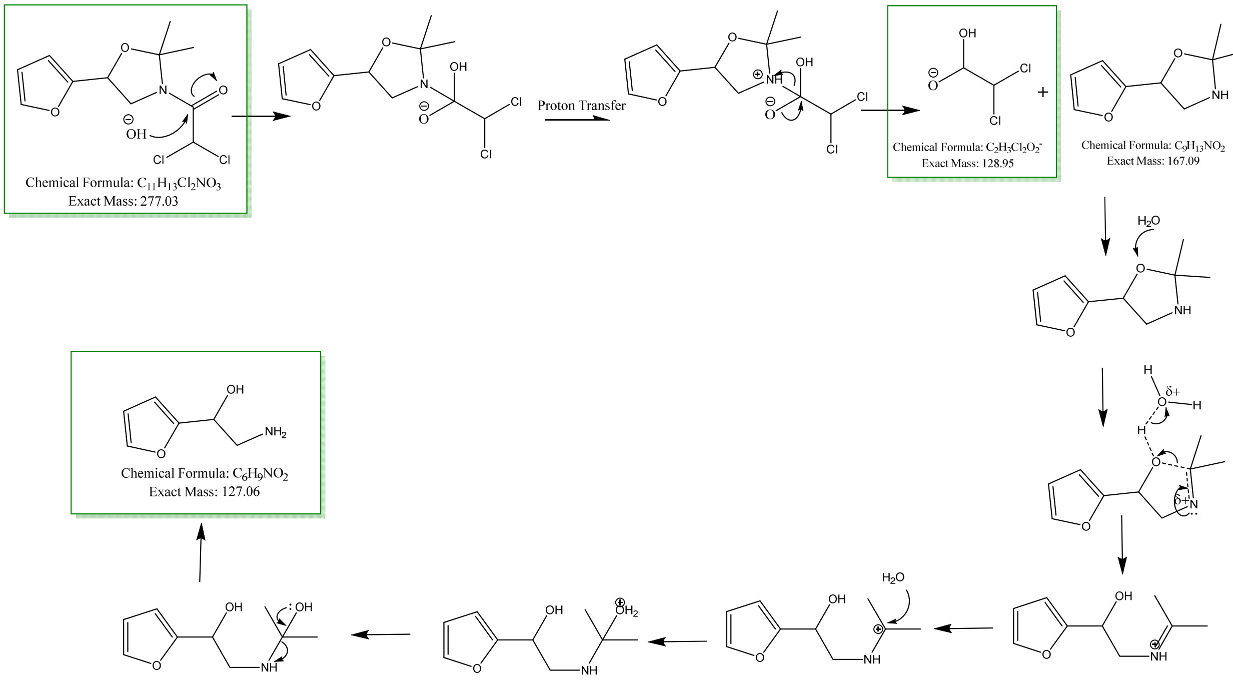

Scheme S4: Proposed mechanism for base-mediated furilazole hydrolysis which yields Furil-127 and dichloroacetate. Ring opening is analogous to that proposed by Fife (1967)[9]

Acid-Mediated Hydrolysis

AD-67: AD 171 was detected as a product of acid-mediated hydrolysis (Figure S10), and a well-documented mechanism involving ring opening and subsequent H_2O addition is proposed (Scheme S6).

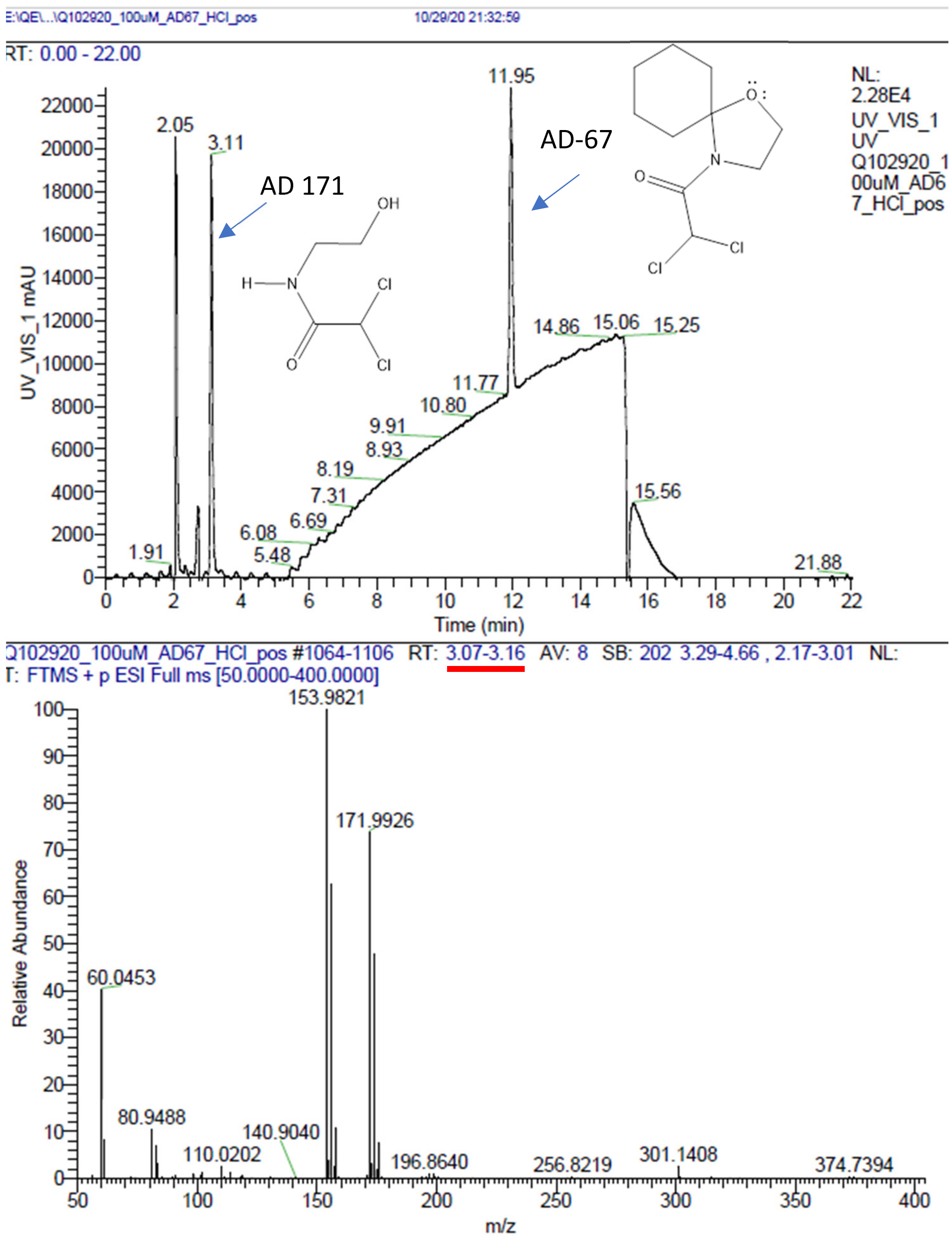

Figure S10: LC chromatogram and Orbitrap MS spectrum of AD-67 acid-mediated hydrolysis product AD-171

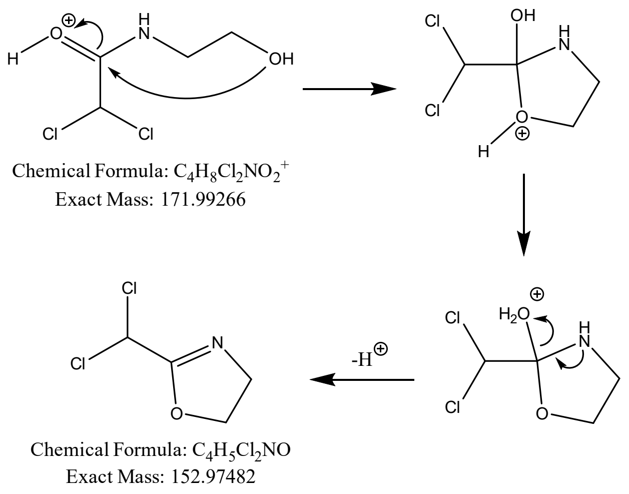

Scheme S5: Proposed mechanism for formation of 153.9821 m/z peak as a result of ionization

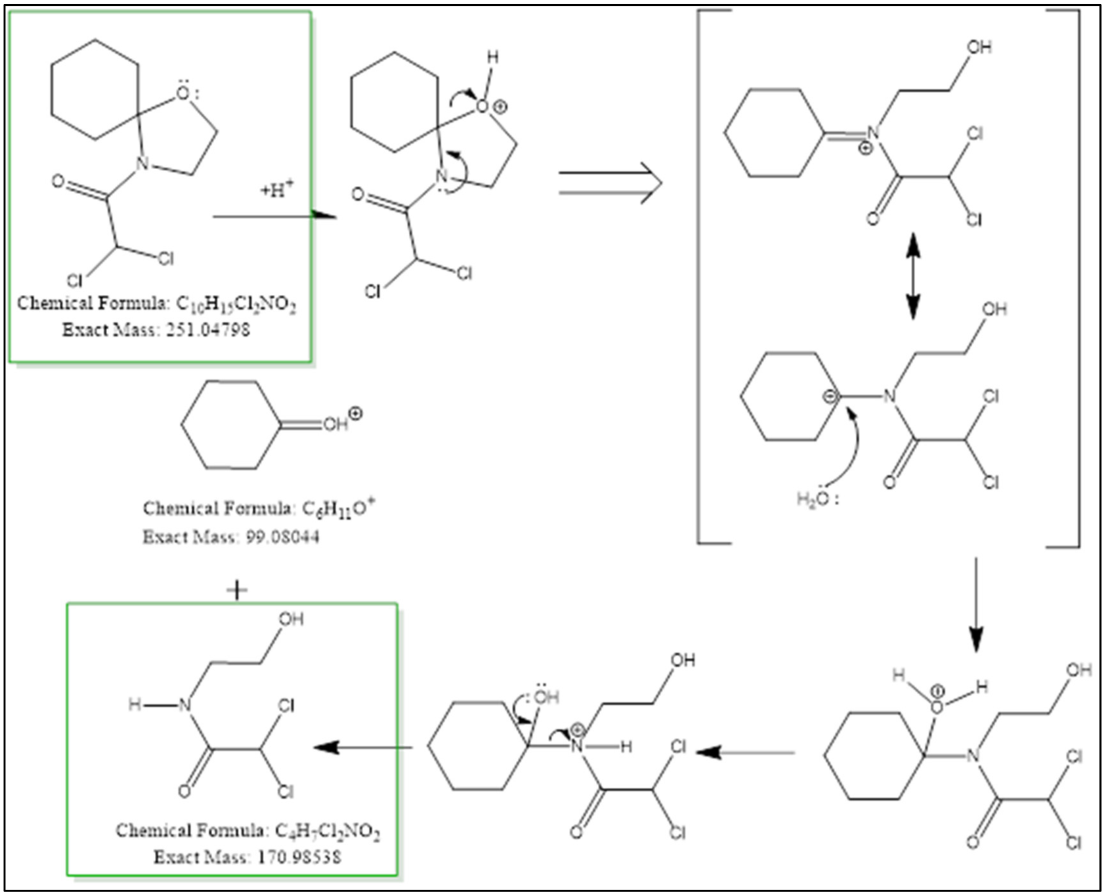

Scheme S6: Proposed mechanism for acid-mediated hydrolysis of AD-67. The highlighted compounds were observed via Orbitrap mass spectrometry.

Furilazole: Furil-237 was detected as a product of furilazole acid-mediated hydrolysis, which we propose follows the same mechanism as AD-67 acid-mediated hydrolysis. AD-67 and furilazole are the only dichloroacetamide safeners which contain an oxazolidine moiety, which is key for the proposed mechanism. An authentic reference standard was not available for Furil-237.

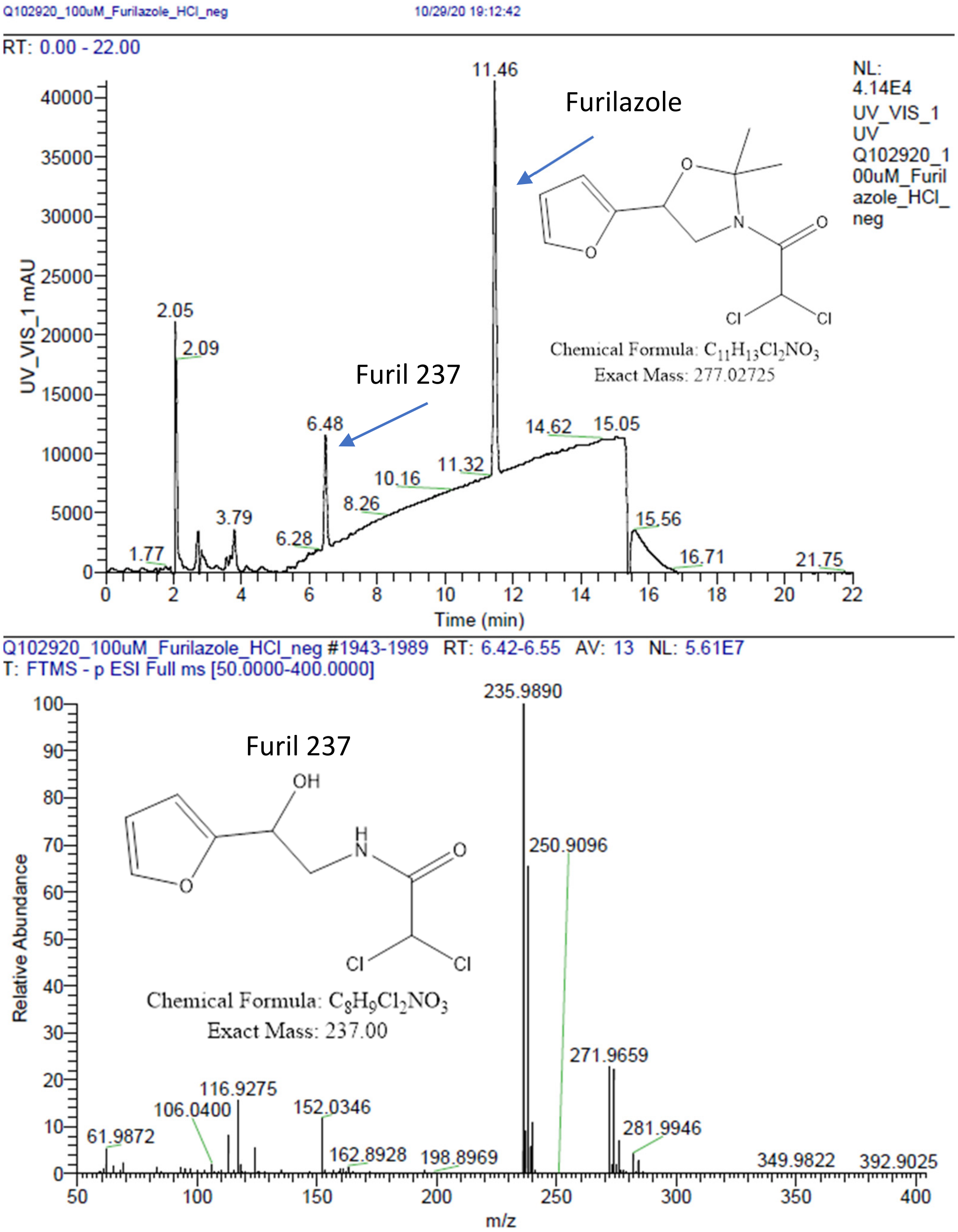

Figure S11: LC chromatogram and HRMS spectrum of furilazole acid-mediated hydrolysis product Furil-237. Note the clear dichloro isotope pattern in the HRMS spectrum.

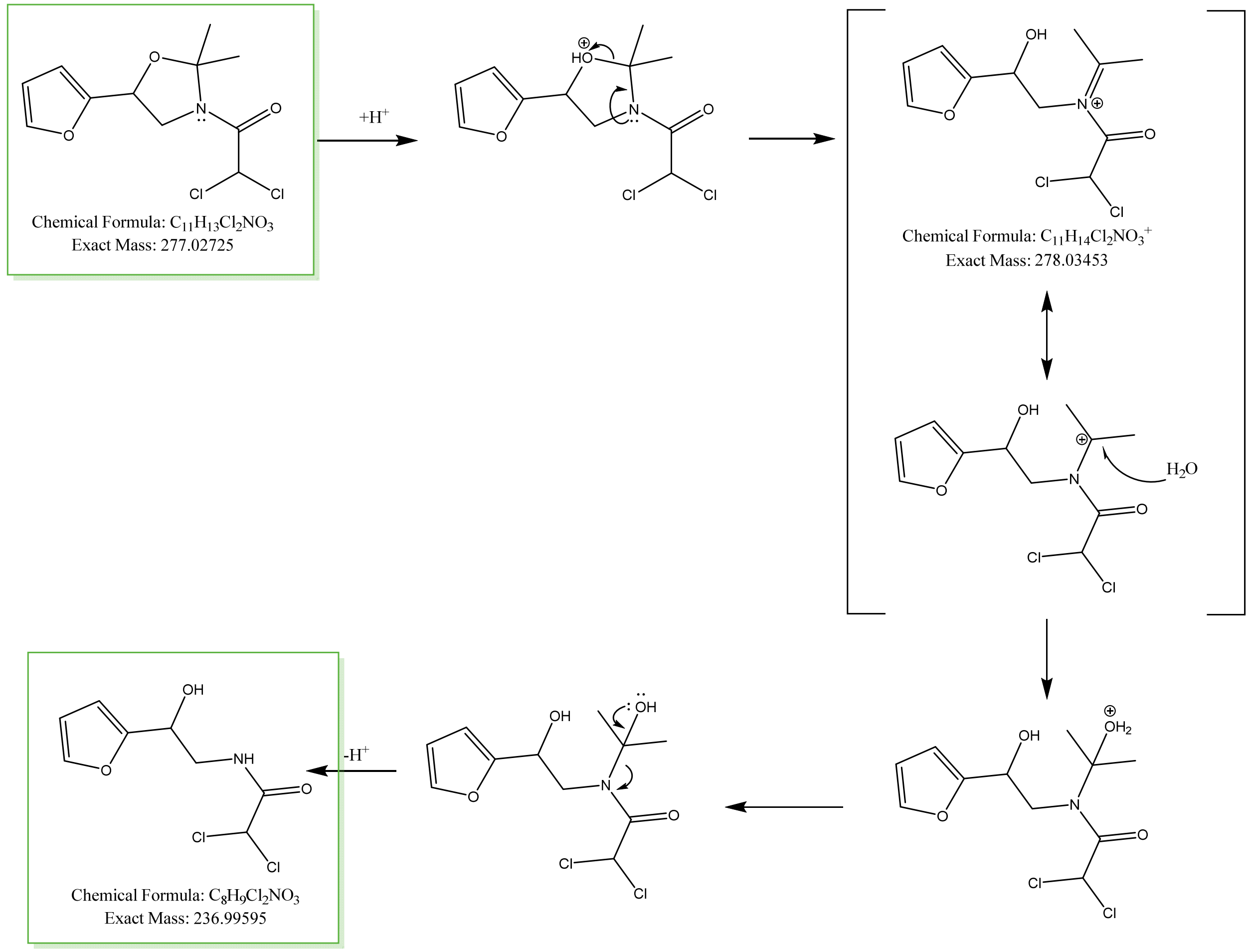

Scheme S7: Proposed mechanism for acid-mediated hydrolysis of furilazole. The highlighted compounds were observed via Orbitrap mass spectrometry.

<u>Benoxacor:</u> Products of acid-mediated benoxacor hydrolysis were not analyzed via mass spectrometry, but their retention times and UV spectra are identical to those of 3-methyl-3,4-dihydro-2H-1,4-benzoxazine and dichloroacetate observed in the basic system. We suggest that the same products are produced via both acid-mediated and base-mediated benoxacor hydrolysis.

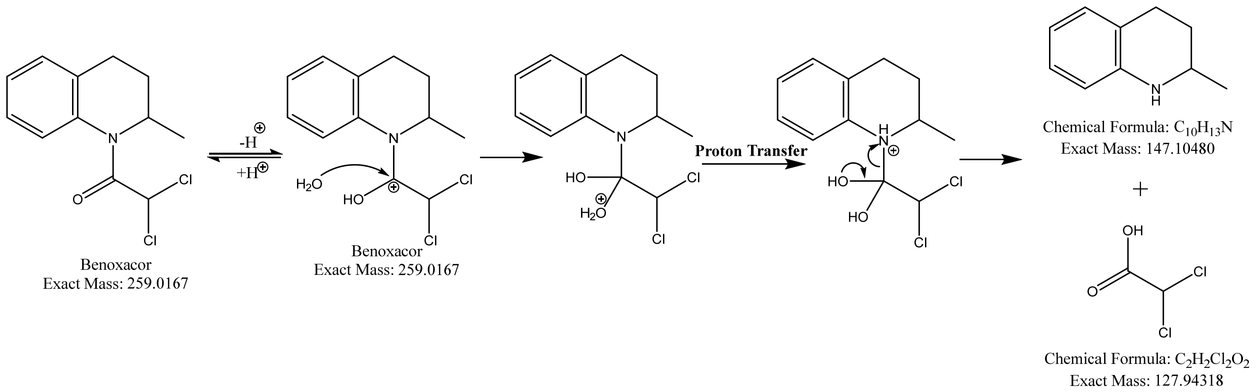

Scheme S8: Proposed mechanism for acid-mediated benoxacor hydrolysis which yields 3-methyl-3,4-dihydro-2H-1,4-benzoxazine and dichloroacetate.

Dichlormid: Dichlormid is the only dichloroacetamide safener that does not undergo acid-catalyzed hydrolysis.

REFERENCES

(1) Van Der Pijl, F.; Van Delft, F. L.; Rutjes, F. P. J. T. Synthesis and Functionalization of Bicyclic N,O-Acetal Scaffolds from Furfural. *Bioorganic Med. Chem.* **2015**, *23* (11), 2721–2729. https://doi.org/10.1016/j.bmc.2014.12.045.

(2) Kral, A.; Pflug, N.; McFadden, M.; LeFevre, G.; Sivey, J.; Cwiertny, D. Photochemical Transformations of Dichloroacetamide Safeners. *Environ. Sci. Technol.* **2019**, *53* (12), 6738–6746.

(3) Hemond, H. F.; Fechner, E. J. *Chemical Fate and Transport in the Environment*, Third.; Elsevier Inc.: Waltham, MA, 2015.

(4) Hamer, W. J.; Wu, Y.-C. Osmotic Coefficients and Mean Activity Coefficients of Uni-Univalent Electrolytes in Water at 25 ° C. *J. Phys. Chem. Ref. Data* **1972**, *1* (4), 1047–1099.

(5) Schwarzenbach, R. P.; Gschwend, P. M.; Imboden, D. M. *Environmental Organic Chemistry*, Third.; John Wiley and Sons, Inc: Hoboken, New Jersey, 2016.

(6) Masbou, J.; Drouin, G.; Payraudeau, S.; Imfeld, G. Carbon and Nitrogen Stable Isotope Fractionation during Abiotic Hydrolysis of Pesticides. *Chemosphere* **2018**, *213* (1), 368–376. https://doi.org/10.1016/j.chemosphere.2018.09.056.

(7) Meikle, R. W.; Youngson, C. R. The Hydrolysis Rate of Chlorpyrifos, O-O-Diethyl O-(3,5,6-Trichloro-2-Pyridyl) Phosphorothioate, and Its Dimethyl Analog, Chlorpyrifos-Methyl, in Dilute Aqueous Solution. *Arch. Environ. Contam. Toxicol.* **1978**, *7* (1), 13–22. https://doi.org/10.1007/BF02332034.

(8) Schymanski, E. L.; Jeon, J.; Gulde, R.; Fenner, K.; Ruff, M.; Singer, H. P.; Hollender, J. Identifying Small Molecules via High Resolution Mass Spectrometry: Communicating Confidence. *Environ. Sci. Technol.* **2014**, *48* (4), 2097–2098. https://doi.org/10.1021/es5002105.

(9) Fife, T. H.; Hagopian, L. Oxazolidine Hydrolysis. The Participation of Solvent and Buffer in Ring Opening. *J. Am. Chem. Soc.* **1968**, *90* (4), 1007–1014. https://doi.org/10.1021/ja01006a028.

(10) Aubé, J. A New Twist on Amide Solvolysis. *Angew. Chemie - Int. Ed.* **2012**, *51* (13), 3063–3065. https://doi.org/10.1002/anie.201108173.
